# Engineered Cell‐Derived Microparticles Bi_2_Se_3_/DOX@MPs for Imaging Guided Synergistic Photothermal/Low‐Dose Chemotherapy of Cancer

**DOI:** 10.1002/advs.201901293

**Published:** 2019-12-12

**Authors:** Dongdong Wang, Yuzhu Yao, Junkai He, Xiaoyan Zhong, Basen Li, Shiyu Rao, Haiting Yu, Shuaicheng He, Xiaoyu Feng, Tuo Xu, Bin Yang, Tuying Yong, Lu Gan, Jun Hu, Xiangliang Yang

**Affiliations:** ^1^ National Engineering Research Center for Nanomedicine College of Life Science and Technology Huazhong University of Science and Technology Wuhan 430074 P. R. China; ^2^ Department of Radiology Tongji Hospital Tongji Medical College Huazhong University of Science and Technology Wuhan 430074 P. R. China

**Keywords:** cell‐derived microparticles, dual‐modal imaging, electroporation, membrane fusion, synergistic therapy

## Abstract

Cell‐derived microparticles, which are recognized as nanosized phospholipid bilayer membrane vesicles, have exhibited great potential to serve as drug delivery systems in cancer therapy. However, for the purpose of comprehensive therapy, microparticles decorated with multiple therapeutic components are needed, but effective engineering strategies are limited and still remain enormous challenges. Herein, Bi_2_Se_3_ nanodots and doxorubicin hydrochloride (DOX) co‐embedded tumor cell‐derived microparticles (Bi_2_Se_3_/DOX@MPs) are successfully constructed through ultraviolet light irradiation‐induced budding of parent cells which are preloaded with Bi_2_Se_3_ nanodots and DOX via electroporation. The multifunctional microparticles are obtained with high controllability and drug‐loading capacity without unfavorable membrane surface destruction, maintaining their excellent intrinsic biological behaviors. Through membrane fusion cellular internalization, Bi_2_Se_3_/DOX@MPs show enhanced cellular internalization and deepened tumor penetration, resulting in extreme cell damage in vitro without considering endosomal escape. Because of their distinguished photothermal performance and tumor homing target capability, Bi_2_Se_3_/DOX@MPs exhibit admirable dual‐modal imaging capacity and outstanding tumor suppression effect. Under 808 nm laser irradiation, intravenous injection of Bi_2_Se_3_/DOX@MPs into H22 tumor‐bearing mice results in remarkably synergistic antitumor efficacy by combining photothermal therapy with low‐dose chemotherapy in vivo. Furthermore, the negligible hemolytic activity, considerable metabolizability, and low systemic toxicity of Bi_2_Se_3_/DOX@MPs imply their distinguished biocompatibility and great potential for tumor theranostics.

## Graphical abstract

Multifunctional cell‐derived microparticles Bi_2_Se_3_/DOX@MPs are successfully fabricated via electroporation with high efficiency and controllability for computed tomography and photoacoustic imaging guided synergistic photothermal/low‐dose chemotherapy. The obtained Bi_2_Se_3_/DOX@MPs show enhanced intracellular uptake through membrane fusion mediated cellular internalization, which not only strengthens their cytotoxicity by avoiding endosomal trapping, but also deepens their penetration in H22 3D tumor spheroids.

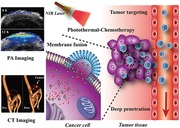

## 1 Introduction

Cell‐derived microparticles (MPs) are phospholipid bilayer membrane vesicles that are secreted by budding from various cell types in response to endogenous or exogenous stimuli.^1^ MPs are now recognized as 100–1000 nm in diameter particles containing proteins, lipids, and genetic material (DNAs, RNAs) from parent cells, which play important roles in intercellular communication.^2^, ^3^, ^4^ Moreover, the capacity of transferring bioactive molecules may allow MPs to be used as potential drug delivery systems (DDS) in cancer therapy.^5^, ^6^, ^7^ During the past few years, excellent progresses have been made in the research of MPs DDS for their great potential in cancer therapy.^8^, ^9^, ^10^, ^11^, ^12^ For example, MPs loaded with different drugs were successfully applied in chemotherapy,^13^, ^14^, ^15^ immunotherapy,^16^, ^17^, ^18^ or gene therapy^19^ of cancers, which have been proved to be effective treatments. MPs DDS exhibit multiple advantages over existing synthetic DDS due to their endogenous origin, such as less immunogenic, long blood circulation ability, and overcoming physiological barrier.^20^, ^21^, ^22^, ^23^, ^24^, ^25^, ^26^, ^27^, ^28^ Besides, the inherent tumor homing target capability without any modification of tumor cell derived MPs also have drawn attention of researchers.^29^, ^30^, ^31^, ^32^ More importantly, the remarkable enhanced cellular internalization of MPs DDS can greatly reduce the dose of expensive anticancer drugs, which may also reduce side effects effectively. However, the reason for this enhanced cellular uptake capacity has not been well studied so far.

Inorganic nanoparticles are extremely attractive for extensive cancer therapy applications due to their typical nanostructure and impressive physicochemical properties.^33^, ^34^, ^35^, ^36^ Combining with traditional method for tumor treatment, enhanced therapeutic effect can usually be reached by improved tumor targeting and synergistic effect of comprehensive therapy. Compared with monotherapeutic methodology, comprehensive therapy and theranostic nanoplatform based on inorganic functional nanoparticles can overcome the therapeutic dilemmas of multifactorial nature of cancer.^37^ For example, photothermal therapy (PTT) takes advantage of the sensibility of cancer cells toward hyperthermia to induce apoptosis, which can kill cancer cells directly or increase their susceptibility to chemotherapy to reduce the dose of drugs or overcome multidrug resistance.^38^, ^39^, ^40^ As an excellent drug delivery system, MPs integrated with functional inorganic nanoparticles will provide a new exciting strategy for tumor treatment. So far, substantial efforts have been devoted to design multifunctional MPs DDS,^41^, ^42^, ^43^ by directly decorating inorganic nanoparticles on the surface of cell‐derived MPs^32^, ^44^, ^45^, ^46^ or previously engineering the parent cells with nanoparticles to obtain MPs with the payloads.^47^, ^48^ Ideal loading methods should not only possess high encapsulation efficiency but also retain the structural and functional integrity of MPs. Direct decoration of MPs is usually a high‐cost and low‐efficiency process with unexpected membrane destroy, which will undoubtedly affect the inherent biological behaviors of MPs.^49^, ^50^ Introducing exogenous functional nanoparticles to parent cells followed by stimuli‐induced budding to generate MPs laden with the payloads seems to be a more acceptable method. However, the loading capacity is largely dependent on the amount of nanoparticles transferred to parent cells, which is a huge obstacle to achieve comparable loading for nonphagocytic cells due to their limited uptake. Constructing multifunctional MPs DDS with high loading capacity in a controllable manner without disrupting the membrane integrity remains a desired goal.

**Scheme 1:**
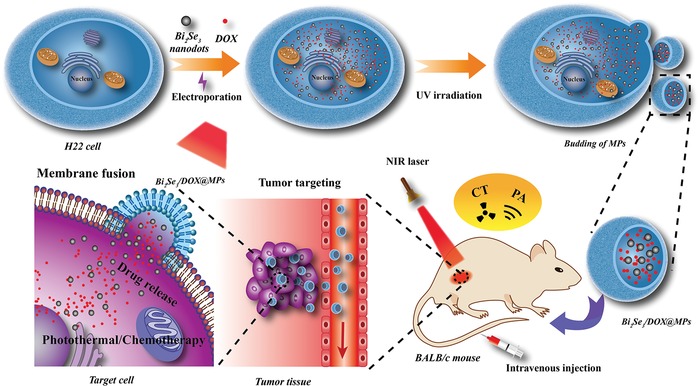
Schematic illustration for fabrication of multifunctional cell‐derived microparticles (Bi_2_Se_3_/DOX@MPs) and their application for CT/PA dual‐modal imaging guided synergistic photothermal/low‐dose chemotherapy.

Aiming to exploit the inherent properties of MPs to design a new generation of multifunctional theranostic vectors with high drug loading capacity and controllability, we proposed an alternative approach in which the parent cells were previously engineered with exogenous components by electroporation instead of active uptake. Electroporation is a well‐known transfer strategy which can deliver DNA, RNA, protein and drugs into target cells with high efficiency by generating transient pores in the plasma membrane.^51^ The cellular uptake of functional inorganic nanoparticles would be increased significantly via electroporation, and the plasma membrane can be reversibly restored by controlling the voltage. In this work, electroporation was firstly employed to transfer PTT agent Bi_2_Se_3_ nanodots and chemo‐drug doxorubicin hydrochloride (DOX) into H22 hepatocellular carcinoma cells. Then the engineered parent cells were irradiated with ultraviolet light to generated MPs laden with both payloads (named Bi_2_Se_3_/DOX@MPs) (**Scheme** 1). Compared with incubation, with this manner, both the drug loading capacity and generation yield of functionalized MPs were dramatically enhanced. In addition, this strategy showed high controllability without an obvious influence on the morphology and functional integrity of MPs as well as their excellent photothermal property. Out of our expectation, the cellular internalization of Bi_2_Se_3_/DOX@MPs showed a membrane fusion manner, which would not only enhance their intracellular uptake, but also strengthen the cytotoxicity in vitro by avoiding endosomal trapping. Furthermore, H22 3D tumor spheroids in vitro mode assessment confirmed the deep tumor penetration capacity of Bi_2_Se_3_/DOX@MPs. Meanwhile, the outstanding dual‐modal computed tomography and photoacoustic (CT/PA) imaging ability of Bi_2_Se_3_/DOX@MPs was utilized to guide the efficient synergistic photothermal‐chemotherapy in vivo at a relatively low‐dose of DOX. The excellent biocompatibility and biodegradability of Bi_2_Se_3_/DOX@MPs is also good for potential clinical translation.

## 2 Results and Discussion

### 2.1 Preparation of Bi_2_Se_3_/DOX@MPs

**Figure 1:**
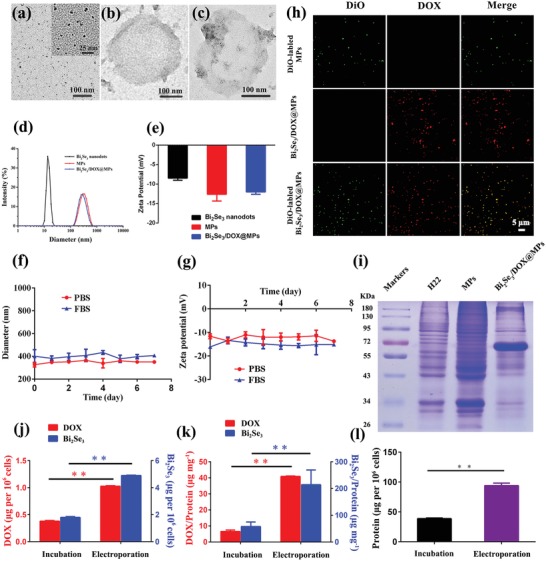
Characterization of Bi_2_Se_3_/DOX@MPs. TEM images of a) Bi_2_Se_3_ nanodots, b) MPs, and c) Bi_2_Se_3_/DOX@MPs. d) Hydrodynamic size distribution and e) zeta potential of Bi_2_Se_3_ nanodots, unloaded MPs and Bi_2_Se_3_/DOX@MPs. Stability of Bi_2_Se_3_/DOX@MPs during storage in PBS and FBS at 4 °C monitored by f) hydrodynamic size and g) zeta potential. h) Fluorescence images of DiO‐labeled MPs, Bi_2_Se_3_/DOX@MPs and DiO‐labeled Bi_2_Se_3_/DOX@MPs. i) SDS‐PAGE protein patterns of H22 cells, unloaded MPs, and Bi_2_Se_3_/DOX@MPs. j) The intracellular content of DOX and Bi_2_Se_3_ (µg 10^−6^ cells) introduced by electroporation or incubation. k) DOX and Bi_2_Se_3_ contents of Bi_2_Se_3_/DOX@MPs generated from donor cells by electroporation or incubation. l) The protein amount of Bi_2_Se_3_/DOX@MPs generated from donor cells by electroporation or incubation.

To prepare Bi_2_Se_3_ and DOX co‐embedded MPs, small‐sized Bi_2_Se_3_ nanodots were firstly synthesized in aqueous solution at room temperature with bovine serum albumin (BSA) as capping agents according to the method reported previously.^52^ Transmission electron microscope (TEM) images in **Figure** 1a indicated that Bi_2_Se_3_ nanodots have uniform spherical morphology with an average size measured to be 2.97 ± 0.38 nm from the statistical analysis. Because of BSA coating, Bi_2_Se_3_ nanodots showed high stability in aqueous solution with a hydrodynamic diameter of 13.46 ± 1.47 nm (Figure 1d), which was bigger than that from TEM due to the formation of hydration layer. From the zeta potential measurement (Figure 1e), Bi_2_Se_3_ nanodots were slightly negatively charged (−8.47 ± 0.55 mV), which would benefit to the following electroporation process to avoid aggregation under the interaction of electric field. By taking advantages of their ultrasmall size, electroneutral surface, and perfect water dispersibility, Bi_2_Se_3_ nanodots accompanied with DOX were directly introduced into in vitro cultured H22 tumor cells via electroporation with high efficiency. The amount of DOX and Bi_2_Se_3_ in tumor cells mediated by electroporation was calculated to be 1.03 ± 0.01 and 4.90 ± 0.03 µg per 10^6^ cells (Figure 1j), which was about 1.7 times higher than that by cellular internalization with traditional co‐incubation method.

Subsequently, Bi_2_Se_3_/DOX@MPs were obtained via irradiating donor cells with ultraviolet light followed by a purification process. From the TEM image of Bi_2_Se_3_/DOX@MPs (Figure 1c, Figure S1b, Supporting Information), a lot of small black dots can be clearly observed in the inner part of Bi_2_Se_3_/DOX@MPs. In contrast, MPs generated by donor cells without Bi_2_Se_3_ internalization look much cleaner (Figure 1b, Figure S1a, Supporting Information). Analysis of the microstructure of Bi_2_Se_3_/DOX@MPs by Scanning TEM energy‐dispersive X‐ray spectroscopy (STEM‐EDS) showed the K, L, M electronic shell of Se and Bi, indicating the successfully packaging of Bi_2_Se_3_ nanodots (Figure S2, Supporting Information). To further identify the loading of DOX, fluorescence analysis was conducted. Under the excitation of 488 nm laser, Bi_2_Se_3_/DOX@MPs showed the characteristic emission bands of DOX while unloaded MPs did not exhibit any typical emission, demonstrating the existence of DOX in Bi_2_Se_3_/DOX@MPs (Figure S3, Supporting Information). Furthermore, fluorescence colocalization analysis was also used to further confirm that DOX was incorporated into the Bi_2_Se_3_/DOX@MPs successfully. MPs and Bi_2_Se_3_/DOX@MPs were labeled with a fluorescent dye 3,3′‐dioctadecyloxacarbocyanine perchlorate (DiO, green) firstly. As shown in Figure 1h, no DOX fluorescent signal (red) could be observed in the DiO labeled‐MPs. In contrast, Bi_2_Se_3_/DOX@MPs exhibited remarkable high DOX fluorescent signals, and almost all the DOX fluorescent signals were colocalized with the fluorescent of DiO.

The hydrodynamic diameter and zeta potential of Bi_2_Se_3_/DOX@MPs were measured to be 356.71 ± 20.41 nm and −12.02 ± 0.59 mV, respectively (Figure 1d), which were the similar as unloaded MPs (360.45 ± 31.81 nm, −12.60 ± 1.75 mV) (Figure 1e), showing that the surface properties of MPs were not influenced by packing of Bi_2_Se_3_ and DOX. To further ensure the membrane integrity, we compared the sodium dodecyl sulfate‐polyacrylamide gel electrophoresis (SDS‐PAGE) protein patterns of H22 cells, MPs generated from H22 cells without drug loading and Bi_2_Se_3_/DOX@MPs. As shown in Figure 1i, the membrane proteins of the three samples were almost the same as expected, except the particular protein band between 55 and 72 kDa for Bi_2_Se_3_/DOX@MPs which was mostly contributed to Bi_2_Se_3_ surface‐coated BSA (66 kDa). Within 7 days' storage in phosphate‐buffered saline (PBS) or fetal bovine serum (FBS) at 4 °C, the size and zeta potential of Bi_2_Se_3_/DOX@MPs showed no remarkable change (Figure 1f,g), indicating their good stability. Furthermore, the cumulative amounts of DOX released from Bi_2_Se_3_/DOX@MPs in pH 7.4 PBS buffer at 37 °C was only 31.95 ± 3.52% after 48 h incubation, showing the membrane integrity and good stability of Bi_2_Se_3_/DOX@MPs (Figure S4, Supporting Information).

As mentioned above, by the manner of electroporation, the intracellular amount of DOX and Bi_2_Se_3_ nanodots was much higher than that by direct endocytosis. Consequently, after UV irradiation, the amount of DOX and Bi_2_Se_3_ in Bi_2_Se_3_/DOX@MPs generated from donor cells by electroporation (40.89 ± 0.26 and 214.48 ± 54.92 µg per mg of protein) was about 5.2 and 2.7 times higher than that by incubation (6.59 ± 0.89 and 57.93 ± 17.09 µg per mg of protein). The drug loading capacity could be controlled by voltage and drug feeding amount in our strategy. As it was illustrated in Figure S5 in the Supporting Information, the optimized voltage was 300 V. Higher voltage will result in irreversible damage to cells, which further reduce the yield of drug‐loaded MPs and drug loading capacity. At the optimized condition, the loading amount of both DOX and Bi_2_Se_3_ reached a maximum value. This value was increased with the feeding amount of DOX and Bi_2_Se_3_ (Figure S6, Supporting Information). The difference between batches was almost negligible, indicating the high controllability of electroporation manner.

Generally, the membrane protein is used for the quantification of MPs. It was worth noting that the total amount of protein for Bi_2_Se_3_/DOX@MPs generated from donor cells by electroporation was also significantly higher than that by incubation, indicating that not only higher encapsulation payload of therapeutic cargoes in each MPs but also enhanced yield of Bi_2_Se_3_/DOX@MPs were obtained by electroporation (Figure 1l), which is extremely important for further bioapplications.

### 2.2 Photothermal Property of Bi_2_Se_3_/DOX@MPs

**Figure 2:**
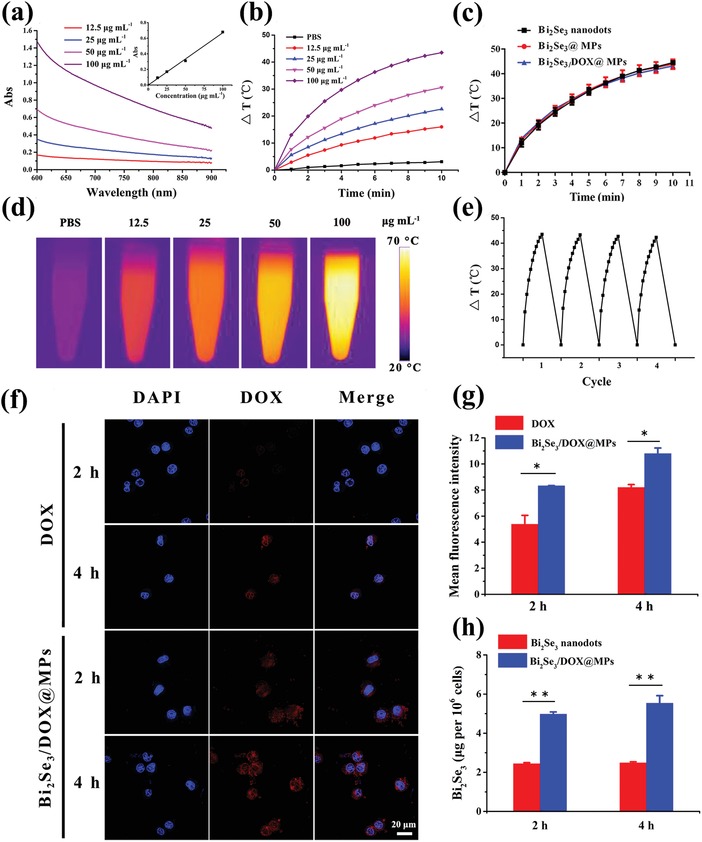
Photothermal properties and enhanced cell uptake of Bi_2_Se_3_/DOX@MPs. a) UV–vis–NIR absorption spectra and b) temperature elevation curves of Bi_2_Se_3_/DOX@MPs at different concentrations. c) Comparison on temperature elevation of Bi_2_Se_3_ nanodots, Bi_2_Se_3_@MPs and Bi_2_Se_3_/DOX@MPs at the same concentration under NIR irradiation. d) Infrared thermal images of Bi_2_Se_3_/DOX@MPs at different concentrations under NIR irradiation. e) Photothermal conversion cycling study of Bi_2_Se_3_/DOX@MPs under NIR irradiation. f) CLSM images and g) mean fluorescence intensity of H22 cells respectively incubated with free DOX and Bi_2_Se_3_/DOX@MPs for 2 and 4 h. h) The containing amount of Bi_2_Se_3_ in H22 cells respectively incubated with Bi_2_Se_3_ nanodots and Bi_2_Se_3_/DOX@MPs for 2 and 4 h.

To assess the NIR photothermal performance, UV–vis–NIR absorption spectra of Bi_2_Se_3_/DOX@MPs was carried out firstly. Bi_2_Se_3_/DOX@MPs showed a broad absorbance in NIR region (**Figure** 2a). In particular, absorption intensity at 808 nm increased linearly with Bi_2_Se_3_ concentrations (Figure 2a, inset), indicating that Bi_2_Se_3_ is responsible for the NIR absorbance. Upon 808 nm irradiation at 1.5 W cm^−2^ for 10 min, Bi_2_Se_3_/DOX@MPs at different Bi_2_Se_3_ concentrations could induce obvious temperature increase as shown in Figure 2b. The temperature variation exhibited a Bi_2_Se_3_ concentration‐dependent manner. For instance, a temperature elevation of 16 °C was observed with Bi_2_Se_3_ concentration at 12.5 µg mL^−1^, while a higher value of 43.5 °C was monitored at a higher concentration of 100 µg mL^−1^. In contrast, PBS showed negligible temperature change under the same irradiation, proving the excellent photothermal property of Bi_2_Se_3_/DOX@MPs. Infrared thermographs of the samples containing Bi_2_Se_3_/DOX@MPs at various concentrations under NIR laser irradiation were also recorded (Figure 2d), which were consistent with the result of temperature elevation curves. We compared the photo‐induced temperature increase of Bi_2_Se_3_ nanodots, Bi_2_Se_3_@MPs and Bi_2_Se_3_/DOX@MPs suspensions at the different Bi_2_Se_3_ concentrations. As it can be seen in Figure 2c and Figure S7 in the Supporting Information, all the three samples exhibited similar temperature rises curves at each given concentration, indicating that the MPs and the loading of DOX had negligible influences on the photothermal performance of Bi_2_Se_3_. It should be noted that there was negligible change in the temperature elevation after four cycles of NIR laser irradiation, indicating the excellent photothermal stability of Bi_2_Se_3_/DOX@MPs (Figure 2e). Hence, they can be considered as a proper candidate for photothermal therapy of cancer.

### 2.3 Intracellular Uptake of Bi_2_Se_3_/DOX@MPs

As aforementioned, MPs DDS exhibit enhanced cellular internalization due to their endogenous origin. In order to verify the hypothesis, the intracellular uptake capability of Bi_2_Se_3_/DOX@MPs in H22 cells was evaluated using confocal laser fluorescence microscopy (CLSM) and flow cytometry. H22 cells were incubated with free DOX, Bi_2_Se_3_ nanodots and Bi_2_Se_3_/DOX@MPs for 2 and 4 h, respectively. As expected, Bi_2_Se_3_/DOX@MP‐treated cells exhibited a much higher DOX fluorescence compared to the slight fluorescence intensity of the free DOX‐treated cells (Figure 2f). Moreover, flow cytometry and atomic fluorescence spectrometer were used to further quantify the cellular uptake of free DOX, Bi_2_Se_3_ nanodots and Bi_2_Se_3_/DOX@MPs, respectively. The mean fluorescence intensity (MFI) of Bi_2_Se_3_/DOX@MP‐treated cells calculated from flow cytometric assay (Figure S8, Supporting Information) was 1.55‐ (2 h) and 1.32‐times (4 h) of DOX‐treated cells (Figure 2g), respectively. Furthermore, to demonstrate the cellular uptake of Bi_2_Se_3_/DOX@MPs directly, TEM images of Bi_2_Se_3_/DOX@MP‐treated cells were exhibited in Figure S9 in the Supporting Information, the small black dots from Bi_2_Se_3_ can be clearly observed in the cells. Besides, a visualized signals caused by heavy metals bismuth was clearly visible in the dark‐field optical microscopy images of Bi_2_Se_3_/DOX@MP‐treated cells, while the control H22 cells without Bi_2_Se_3_/DOX@MPs showed no obvious signals (Figure S10, Supporting Information). And quantified by atomic fluorescence spectrophotometer, as shown in Figure 2h, the intracellular amount of Bi_2_Se_3_ for Bi_2_Se_3_/DOX@MP‐treated cells was 2.03‐ (2 h) and 2.23‐times (4 h) of Bi_2_Se_3_ nanodot‐treated cells. It was obvious that the assistance of MPs significantly enhanced the cellular internalization of DOX and Bi_2_Se_3_ nanodots. Not only that, more DOX was observed inside the nuclei when MPs were used as drug delivery carriers even at 2 h incubation. The cellular internalization ability of Bi_2_Se_3_/DOX@MPs by different cell lines was verified by flow cytometry analysis. As shown in Figure S11 in the Supporting Information, H22 cells showed the highest cellular internalization owing to the parent source of Bi_2_Se_3_/DOX@MPs.

### 2.4 Cellular Internalization Pathway of Bi_2_Se_3_/DOX@MPs

**Figure 3:**
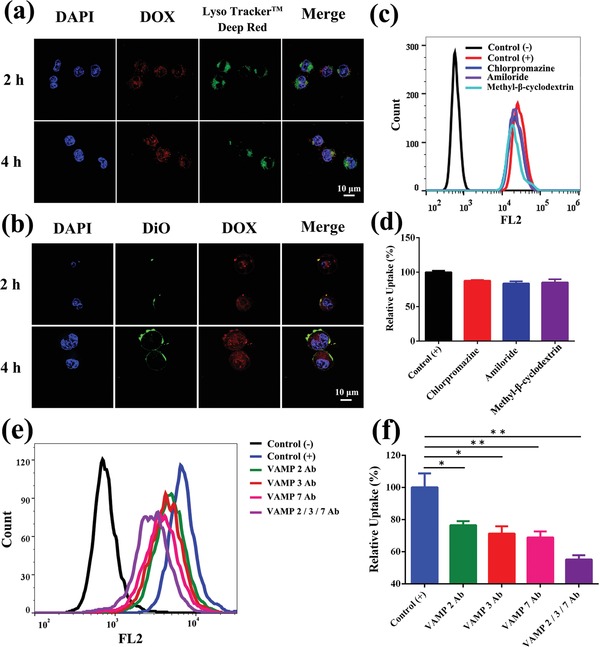
Cellular internalization pathway of Bi_2_Se_3_/DOX@MPs. CLSM images of H22 cells incubated with a) Bi_2_Se_3_/DOX@MPs and b) DiO‐labeled Bi_2_Se_3_/DOX@MPs for 2 and 4 h. Cell nucleus were labeled with DAPI (blue) in both (a) and (b). Lysosomes were labeled with LysoTracker Deep Red (green) in (a). The concentration of DOX was fixed at 1 µg mL^−1^. c) Flow cytometric profile and d) relative cellular uptake of Bi_2_Se_3_/DOX@MPs in the presence of specific endocytosis inhibitors. e) Flow cytometric profile and f) relative cellular uptake of Bi_2_Se_3_/DOX@MPs before or after blocking by VAMP 2 Ab, VAMP 3 Ab, VAMP 7 Ab, VAMP 2/3/7 Ab.

To explore the possible reason of the enhanced cell uptake of Bi_2_Se_3_/DOX@MPs, the cellular internalization pathway was studied. LysoTracker Deep Red was used for lysosome staining. As shown in **Figure** 3a, after 2 and 4 h incubation, the fluorescence signals of DOX (red) and LysoTracker Deep Red (green) showed negligible colocalization in cells. Moreover, the addition of various endocytic inhibitors including chlorpromazine (clathrin‐mediated inhibitor), amiloride (macropinocytosis inhibitor), and methyl‐β‐cyclodextrin (caveolae‐mediated inhibitor) led to a few reductions in the uptake of Bi_2_Se_3_/DOX@MPs (Figure 3c,d). These results demonstrated that endocytosis was not the main pathway of the cellular internalization of Bi_2_Se_3_/DOX@MPs. To further confirm our result, the external phospholipid bilayer membrane of Bi_2_Se_3_/DOX@MPs was labeled with green fluorescence DiO to track their intracellular behavior. When cells were incubated with DiO labled‐Bi_2_Se_3_/DOX@MPs for 2 h, intensive green fluorescence signals were mainly emerged on cell surface, while the red fluorescence signals of DOX were widely distributed within the cells (Figure 3b). It means that the DOX were released from Bi_2_Se_3_/DOX@MPs and the external phospholipid bilayer membrane of Bi_2_Se_3_/DOX@MPs was still in step with cell membrane in the process of cellular internalization. After 4 h incubation, both the green and red fluorescence signals were enhanced. Meanwhile, the green fluorescence signals further extended along the whole cell membrane, indicating a typical membrane fusion process (Figure 3b). As previously reported, membrane fusion is also a temperature‐dependent process.^53^ It was found that the cellular uptake of Bi_2_Se_3_/DOX@MPs at 4 °C was only about 36.92 ± 0.41% compared to that at 37 °C (Figure S12, Supporting Information). Human hepatocellular carcinoma Bel7402 cell, a kind of adherent cell facilitating observation of subcellular behavior, was also used to study the cellular internalization pathway of Bi_2_Se_3_/DOX@MPs. As shown in Figure S13 in the Supporting Information, a similar result was obtained. A few green fluorescence signals within cells over time were ascribed to the free diffusion of DiO. All these results indicated that the cellular internalization of Bi_2_Se_3_/DOX@MPs was mainly mediated through membrane fusion, which was extremely beneficial to cell uptake of Bi_2_Se_3_/DOX@MPs.

The mechanism of this membrane fusion process has also been studied. It was reported that soluble *N*‐ethylmaleimide‐sensitive factor attachment protein receptor (SNARE) proteins played important role in membrane fusion.^54^, ^55^ SNARE proteins include a series of vesicle‐membrane fusion‐related proteins. To clarify whether SNARE proteins were involved in the cellular uptake of Bi_2_Se_3_/DOX@MPs, vesicle‐associated membrane protein 2, 3, or 7 (VAMP 2, VAMP 3 and VAMP 7) antibody (Ab) was used to pretreat Bi_2_Se_3_/DOX@MPs and then determined the cellular uptake of Bi_2_Se_3_/DOX@MPs by flow cytometry. As shown in Figure 3e,f, the relative cellular uptake of VAMP 2, 3, or 7 Ab‐treated Bi_2_Se_3_/DOX@MPs was only about 76.50%, 71.32%, or 68.87% of that of nontreated Bi_2_Se_3_/DOX@MPs, respectively. Moreover, when Bi_2_Se_3_/DOX@MPs was co‐treated by VAMP 2, 3, and 7 Ab, their relative cellular uptake was as low as 55.19%. These data suggest that these membrane fusion‐related SNARE proteins are highly correlated with the enhanced cellular uptake of Bi_2_Se_3_/DOX@MPs. Besides, it was reported that calcium ions are involved in the SNARE protein‐related membrane fusion process, and the fusion will be enhanced in response to Ca^2+^ increase.^54^ As shown in Figure S14 in the Supporting Information, after adding 200 × 10^−6^ m Ca^2+^, the relative cellular uptake of Bi_2_Se_3_/DOX@MPs was significantly enhanced, further supporting the notion that SNARE proteins on Bi_2_Se_3_/DOX@MPs might be involved in the cellular internalization.

### 2.5 Deep Penetration in 3D Tumor Spheroids

**Figure 4:**
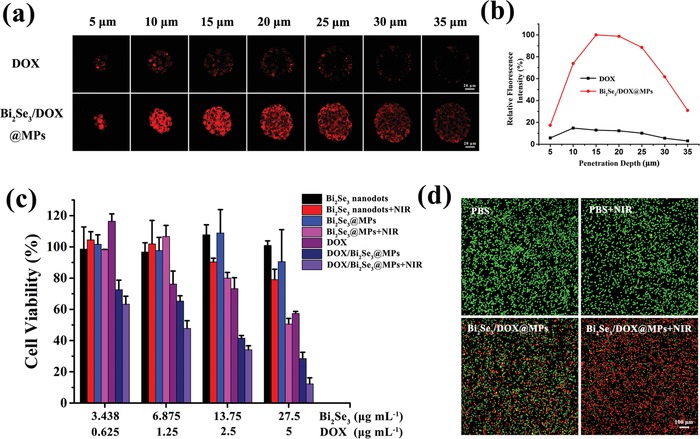
In vitro penetration and cytotoxicity of Bi_2_Se_3_/DOX@MPs. a) Z‐stack CLSM images and b) relative fluorescence intensity of DOX into H22 3D tumor spheroids treated with free DOX and Bi_2_Se_3_/DOX@MPs for 4 h. c) In vitro cytotoxicity of free DOX, Bi_2_Se_3_ nanodots, Bi_2_Se_3_/@MPs and Bi_2_Se_3_/DOX@MPs with or without NIR irradiation at different concentrations on H22 cells after 24 h incubation. d) Fluorescence images of H22 cells treated with PBS and Bi_2_Se_3_/DOX@MPs with or without NIR irradiation. The living cells were stained with calcein‐AM (green) and the dead cells were stained with PI (red).

As we all know, the therapeutic efficacy is usually hampered by the limited penetration depth of nanocarriers in tumor.^56^ Consequently, the penetration ability for Bi_2_Se_3_/DOX@MPs into H22 tumor was estimated using H22 3D tumor spheroids in vitro mode as an in vivo like tumor.^57^ After incubating H22 3D tumor spheroids with free DOX and Bi_2_Se_3_/DOX@MPs for 4 h respectively, the fluorescence signals of DOX were observed by CLSM. As shown in **Figure** 4a, the penetration of free DOX was mostly limited to the peripheral cell layers of the tumor spheroids. In contrast, Bi_2_Se_3_/DOX@MPs showed deeper delivery of DOX, a strong DOX fluorescence signal was observed even in the center of tumor spheroids at 35 µm depth. Furthermore, the total fluorescence intensity of DOX in each slice in the Z‐stacks of tumor spheroids was semiquantitative analyzed and the results were depicted in Figure 4b. The DOX fluorescence intensity of Bi_2_Se_3_/DOX@MPs treated tumor spheroids with a maximum at 15 µm depth was defined 100% relative fluorescence intensity. It was found that the DOX fluorescence intensity of Bi_2_Se_3_/DOX@MPs treated tumor spheroids were 6.7‐fold and 9.1‐fold higher than that of free DOX treated ones at 15 and 35 µm depth, respectively. The deeper penetration of Bi_2_Se_3_/DOX@MPs in H22 3D tumor spheroids was likely due to the enhanced cellular internalization of Bi_2_Se_3_/DOX@MPs via membrane fusion. While Bi_2_Se_3_/DOX@MPs were uptaken by H22 cells in the surface layer of tumor spheroids, drugs were released within the cells and new generation of drug‐loaded MPs were formed, which could transfer to the inner layer of H22 3D tumor spheroids by domino effect.^5^

### 2.6 In Vitro Synergistic Photothermal‐Chemotherapeutic Efficacy

After conducting the photothermal performance and cellular internalization study of Bi_2_Se_3_/DOX@MPs, we examined the synergistic photothermal‐chemotherapeutic efficacy in vitro on H22 cells. As shown in Figure 4c, after incubated with Bi_2_Se_3_ nanodots or Bi_2_Se_3_@MPs for 24 h, H22 cells exhibited viability higher than 90%, indicating their good biocompatibility. Once loading DOX accompanied with Bi_2_Se_3_ nanodots into MPs, they showed obvious concentration‐dependent cell cytotoxicity. Cell viability was dramatically decreased to 28.4% when the concentration of packed DOX was 5 µg mL^−1^. However, at the same dose, free DOX gives cell viability at 57.4%. Similar result was obtained when comparing the cell viability of Bi_2_Se_3_ nanodots plus NIR and Bi_2_Se_3_@MPs plus NIR treating groups. The former was measured to be 79.1% and the latter was 50.6%. The enhanced cytotoxicity was attributed to the enhanced cellular internalization mediated by MPs. Among all groups, Bi_2_Se_3_/DOX@MPs plus NIR irradiation induced the highest cell damage, demonstrating the synergetic effect of photothermal‐chemotherapy. And the membrane fusion of MPs with recipient cells directly released DOX and Bi_2_Se_3_ nanodots into the cytoplasm, avoiding endosomal trapping, also enhancing the effectiveness of Bi_2_Se_3_/DOX@MPs.^58^ Thus, the low therapeutic dose could be achieved for the efficient cytotoxicity of Bi_2_Se_3_/DOX@MPs in vitro. In addition, as reported before,^5^ drug‐encapsulated MPs may impede drug efflux and induce domino‐like cell killing.

Furthermore, the synergistic therapeutic effect of Bi_2_Se_3_/DOX@MPs in vitro was directly observed via calcein acetoxymethyl ester (calcein‐AM) and propidium iodide (PI) double staining for identifying live (green fluorescence) and dead (red fluorescence) cells. As shown in Figure 4d, compared with NIR illumination group and Bi_2_Se_3_/DOX@MPs treating group, Bi_2_Se_3_/DOX@MPs+NIR group exhibited more obvious tumor cell inhibition. All these results showed the remarkable synergistic photothermal‐chemotherapeutic efficacy of Bi_2_Se_3_/DOX@MPs under NIR irradiation.

### 2.7 In Vivo Hemolytic Activity, Biodistribution, and Photothermal Performance

Hemolytic activity assay was critical to evaluate the hemocompatibility of Bi_2_Se_3_/DOX@MPs under physiological conditions. It was also usually used to study the feasibility of intravenous injection of drugs. The quantitative detection of hemoglobin released from erythrocyte cells was utilized to calculate the hemolytic activity percentage. Erythrocyte cells respectively incubated with deionized (DI) water and physiological saline were used as the positive (+) and negative (−) control. As shown in Figure S15 in the Supporting Information, the released hemoglobin was nearly undetectable when BALB/c mice blood samples were treated with Bi_2_Se_3_/DOX@MPs at different concentrations, indicating their excellent hemocompatibility. The hemolytic activity percentage was as low as 0.52% even at a high Bi_2_Se_3_/DOX@MPs concentration (200 µg mL^−1^), which was much lower than the recognized safe value of 5%. As a result, the negligible hemolytic activity of Bi_2_Se_3_/DOX@MPs had been verified, demonstrating the biosafety and feasibility for them as intravenous injection drugs for in vivo studies.

**Figure 5:**
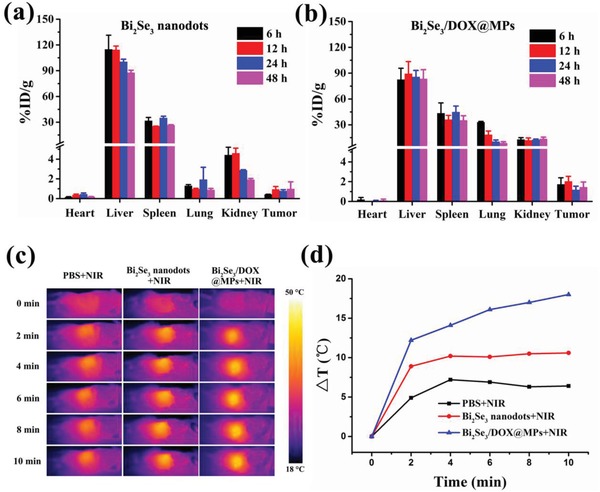
In vivo biodistribution and photothermal performance of Bi_2_Se_3_/DOX@MPs. In vivo biodistribution monitored by Bi element after injecting a) Bi_2_Se_3_ nanodots and b) Bi_2_Se_3_/DOX@MPs intravenously at 6, 12, 24, and 48 h. c) Infrared thermal images H22 tumor‐bearing BALB/c mice injected with PBS, Bi_2_Se_3_ nanodots, Bi_2_Se_3_/DOX@MPs for 12 h with NIR irradiation. d) Corresponding temperature increase in tumor site measured from c.

After intravenous injection of Bi_2_Se_3_/DOX@MPs via tail vein, H22 tumor‐bearing BALB/c mice were sacrificed at different time points (6, 12, 24, 48 h) to examine the biodistribution of the drugs. The containing amount of Bi in tumor tissues and main organs were detected and the results were shown in **Figure** 5b. Due to the usualness accumulation for nanoparticles in the reticuloendothelial system, the liver and spleen were the main target organs. In addition, comparing to the mice treated with Bi_2_Se_3_ nanodots (Figure 5a), those administered with Bi_2_Se_3_/DOX@MPs showed higher lung accumulation, which was due to the sub‐micrometer size of Bi_2_Se_3_/DOX@MPs. It was worth noting that the amount of Bi in tumor region of the mice administered with Bi_2_Se_3_/DOX@MPs was significantly higher than that with Bi_2_Se_3_ nanodots. Drug accumulation reached the highest value at 12 h post injection. Higher DOX fluorescence signals in tumor site for Bi_2_Se_3_/DOX@MP‐treated group could also be observed at 12 h post injection, comparing to that for free DOX group (Figure S16, Supporting Information). The deep tumor penetration and enhanced tumor cell internalization may result in the efficient accumulation of Bi_2_Se_3_/DOX@MPs in tumor tissue. More importantly, Bi_2_Se_3_/DOX@MPs displayed tumor homing target capability that is likely dependent on the proteins of their membrane.^59^ CD54 (ICAM1), a member of the immunoglobulin supergene family, has been reported to play an important role in the tumor targeting of tumor cell‐derived extracellular vesicles.^60^ To clarify whether CD54 was involved in the enhanced tumor accumulation of Bi_2_Se_3_/DOX@MPs, Bi_2_Se_3_/DOX@MPs were pretreated with CD54 Ab and then their in vivo biodistribution was determined at 12 h after intravenous injection. As shown in Figure S17, Supporting Information, pretreatment with CD54 Ab significantly decreased the tumor accumulation of Bi_2_Se_3_/DOX@MPs, confirming the role of CD54 on the MPs in the enhanced tumor accumulation of Bi_2_Se_3_/DOX@MPs. To further explore the effect of proteins on Bi_2_Se_3_/DOX@MPs on the tumor targeting ability, Bi_2_Se_3_/DOX@MPs was treated with 0.25% trypsin‐EDTA (TE) to deplete external proteins on the MPs without affecting their structural integrity.^61^ Consistently, the tumor accumulation of TE‐treated Bi_2_Se_3_/DOX@MPs was significantly reduced by 65.8%, further confirming that the proteins on the MPs contributed to the enhanced tumor accumulation of Bi_2_Se_3_/DOX@MPs. The photothermal performance of Bi_2_Se_3_/DOX@MPs in vivo was also investigated. Based on the result of biodistribution, after 12 h intravenous injection of Bi_2_Se_3_/DOX@MPs, infrared thermal images of tumor‐bearing mice exposed upon 808 nm irradiation for different time were recorded (Figure 5c). The corresponding temperature variation along with irradiation time (Figure 5d) in tumor site was also monitored. As it was shown, Bi_2_Se_3_/DOX@MPs induced a rapid temperature increase in tumor site to 46 °C within 10 min, which was suitable for killing cancer cells by hyperthermia. At the same drug and light dose, Bi_2_Se_3_ nanodots resulted in a less temperature increase, attributing to their relatively poor tumor targeting ability. As a control, the increment of tumor site temperature for the mouse administrated with PBS was only about 7 °C after 10 min illumination of 808 nm laser, demonstrating that light dose used here was safe and reliable for normal tissue. The photothermal performance of Bi_2_Se_3_/DOX@MPs confirmed their potential applications in tumor photothermal therapy.

### 2.8 In Vitro and In Vivo CT/PA Imaging

**Figure 6:**
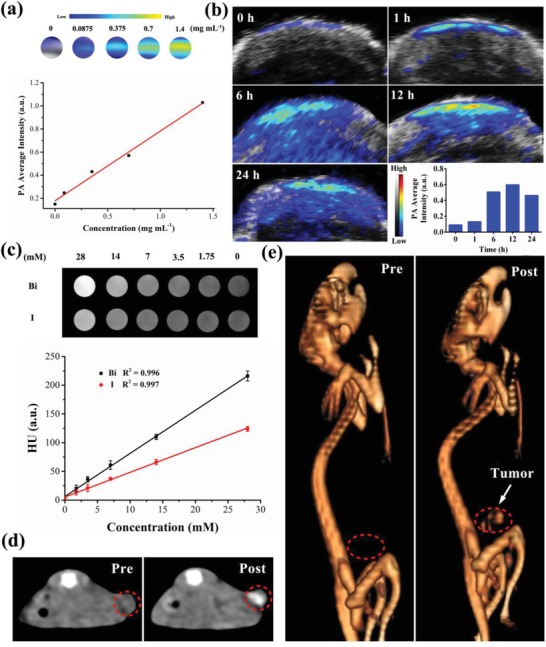
Dual‐modal imaging of Bi_2_Se_3_/DOX@MPs. a) Color‐mapped PA images and measured PA intensity of Bi_2_Se_3_/DOX@MPs at different Bi_2_Se_3_ concentrations. b) In vivo PA imaging of tumor site before and after intravenous injection of Bi_2_Se_3_/DOX@MPs (20 mg kg^−1^) at different time points, corresponded with the average PA intensity. c) CT images and HU value of Bi_2_Se_3_/DOX@MPs and iohexol at different concentrations. d) In vivo transverse section and e) reconstructed 3D CT images of tumor‐bearing mice before and after injection of Bi_2_Se_3_/DOX@MPs.

Multimodal imaging is especially demanded in DDS as it can be used for guiding the therapeutic process, monitoring therapeutic efficacy and reducing extra lesion to normal tissue.^62^ As an emerging imaging modality with high sensitivity and depth imaging, PA imaging has been broadly proposed in cancer diagnosis and therapy.^63^ Bi_2_Se_3_/DOX@MPs can be utilized as a good PA contrast agent for PA imaging because of its high NIR absorbance and efficient photothermal conversion ability. As shown in **Figure** 6a, the PA signal generated by Bi_2_Se_3_/DOX@MPs remarkable enhanced linearly with the increase of Bi_2_Se_3_/DOX@MPs concentrations (Bi_2_Se_3_ concentrations: from 0 to 1.4 mg mL^−1^). Then, H22 tumor‐bearing BALB/c mice were intravenously injected with Bi_2_Se_3_/DOX@MPs to assess its PA imaging in vivo. It was found that PA signal within tumor region increased gradually over time after injection, owing to the accumulation of Bi_2_Se_3_/DOX@MPs in the tumor (Figure 6b). At 12 h post injection, the average PA signal intensity in the tumor site reached to the maximum, which was about 5.6 times higher than that before injection. The PA signal slightly decreased after 24 h post injection at last. As expected, the PA imaging result was in agreement with the biodistribution of Bi_2_Se_3_/DOX@MPs in vivo. In addition, because of X‐ray attenuation ability of bismuth, Bi_2_Se_3_/DOX@MPs can also be used as a promising contrast agent in CT imaging. To test this, Bi_2_Se_3_/DOX@MPs and commercial iohexol at a series of Bi/I concentrations (from 0 to 28 × 10^−3^ m) were imaged in vitro using a CT system. As shown in Figure 6c, an obvious concentration‐dependent CT contrast effect could be observed. The calculated X‐ray absorption coefficient of Bi_2_Se_3_/DOX@MPs was about 7.5 HU per millimolar of Bi, which was remarkably higher than that of iohexol (4.3 HU per millimolar of I). The in vivo CT images of H22 tumor‐bearing mice before and after injection of Bi_2_Se_3_/DOX@MPs were also illustrated in Figure 6d,e. The transverse section and reconstructed 3D CT images exhibited that a noticeable strengthening contrast was observed in the tumor site after injection of Bi_2_Se_3_/DOX@MPs. Thus, the excellent CT/PA imaging performance of Bi_2_Se_3_/DOX@MPs is beneficial to guiding its synergistic photothermal‐chemotherapy in vivo.

### 2.9 In Vivo Antitumor Activity of Synergistic Therapies

**Figure 7:**
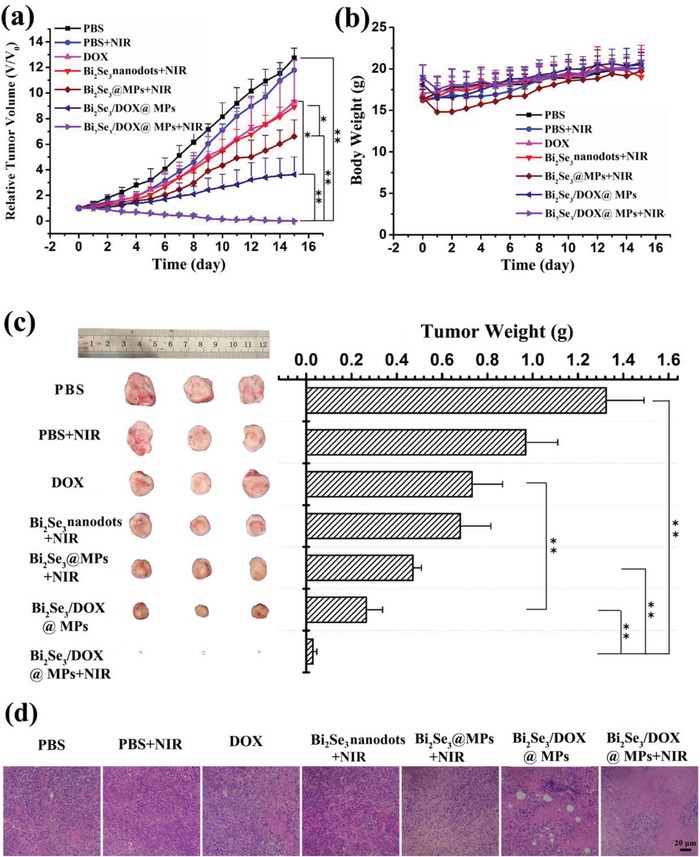
In vivo tumor inhibition of Bi_2_Se_3_/DOX@MPs. Relative a) tumor volumes and b) body weights of H22 tumor‐bearing BALB/c mice after different treating. c) Representative photos and average tumor weight of excised tumors 15 days after treatments. d) H&E staining (200 ×) of the tumor tissues after the indicated treatments.

Encouraged by the performance of synergetic photothermal‐chemotherapeutic efficacy in vitro, the synergistic therapeutic efficacy of Bi_2_Se_3_/DOX@MPs in vivo was evaluated on H22 tumor‐bearing BALB/c mice. When the tumor volume reached 60–80 mm^3^, mice were divided into seven groups stochastically: PBS group, PBS+NIR group, free DOX group, Bi_2_Se_3_ nanodots+NIR group, Bi_2_Se_3_@MPs+NIR group, Bi_2_Se_3_/DOX@MPs group and Bi_2_Se_3_/DOX@MPs+NIR group. The treating groups were received therapeutic dose of 1.2 mg kg^−1^ of DOX and/or 6.6 mg kg^−1^ of Bi_2_Se_3_ by intravenous injection. 12 h post‐injection, the tumor tissue was locally irradiated with 808 nm NIR laser at a power density of 1.5 W cm^−2^ for 10 min. As shown in **Figure** 7a, compared with the control group, free DOX and Bi_2_Se_3_ nanodots plus NIR treating had slight tumor inhibition ability, due to the low therapeutic dose and poor tumor accumulation. Once embedded into MPs, their antitumor activities were appreciable for the multiple advantages of MPs DDS as described above but did not achieve anticipated antitumor effects. It was only the Bi_2_Se_3_/DOX@MPs+NIR group which exhibited significant synergistic photothermal‐chemotherapy effect and reached complete tumor inhibition (tumor inhibition rate was calculated to be 97.73%) without obvious weight loss of mice (Figure 7b). Such synergistic effect could be attributed to the enhanced tumor sensitivity to chemotherapy and cellular uptake of Bi_2_Se_3_/DOX@MPs via elevating the tumor temperature by hyperthermia. Moreover, tumors were excised from mice after 15‐day treatment, the antitumor activity were also verified by photographs and weight of tumors from different groups (Figure 7c). The hematoxylin and eosin (H&E) staining analysis (Figure 7d) showed that most tumor cells were seriously destroyed with their nuclei broken into pieces for Bi_2_Se_3_/DOX@MPs+NIR group.

As is well known, it is a potential threat to health along with the accumulation of metallic nanoparticles in the body.^64^ Therefore, the metabolic of bismuth in major organs at long periods of time (day 3, 7, and 14) after intravenous injection of Bi_2_Se_3_/DOX@MPs were evaluated. It was observed that a considerable decrease of bismuth contents in heart, liver, spleen, lung, and kidney (Figure S18, Supporting Information), respectively. The mechanism of such time‐dependent clearance was attributed to the small size and degradation of Bi_2_Se_3_. According to the H&E staining images of major organs (Figure S19, Supporting Information), no noticeable inflammation or damage was observed in the heart, liver, spleen, lung, and kidney for the mice in the treating group as compared to those in the control groups. In our drug delivery system, the fairly low therapeutic dose of DOX (1.2 mg kg^−1^) also decreased the risk of side effect. Blood biochemistry analysis (ALT, AST, CK, LDH, BUN) was conducted as presented in Figure S20 in the Supporting Information; no obvious toxicity to heart, liver, and kidney was observed. Taken together, the results demonstrated the negligible systemic toxicity of the synergistic treatment in vivo.

## 3 Conclusion

In summary, we have successfully developed a multifunctional cell‐derived MPs drug delivery system (Bi_2_Se_3_/DOX@MPs) for dual‐modal imaging guided synergistic photothermal/low‐dose chemotherapy of cancer. Different from existing strategies for labeling MPs, Bi_2_Se_3_/DOX@MPs were obtained by prepacking Bi_2_Se_3_ nanodots and DOX into donor cells via electroporation followed by ultraviolet light irradiation induced budding, avoiding membrane surface destruction of MPs which can maintain their intrinsic biological behaviors at the maximum extent. In addition, high production yield and drug loading capacity can also be reached. As a result, Bi_2_Se_3_/DOX@MPs exhibit enhanced cellular internalization through membrane fusion, deep penetration in 3D tumor spheroids, admirable tumor targeting, and excellent photothermal performance. They showed extremely lower cell viability (12.3%) in vitro by combining PTT and chemotherapy. Consistently, a relatively high tumor inhibition rate of 97.73% was obtained for Bi_2_Se_3_/DOX@MPs under NIR irradiation even at very low dose of DOX, attributing to the synergistic effect of PTT and chemotherapy. Biogenetic MPs, ultrasmall‐size Bi_2_Se_3_ nanodots, and low‐dose DOX endow our drug delivery system with distinguished biocompatibility and negligible toxicity. Thus, this work is expected to provide new insight into MP‐related DDS for multimodal imaging guided synergistic therapy of cancer.

## 4 Experimental Section

*Materials*: Hydrochloric acid and ethylenediamine were purchased from Tianjin Guangfu Fine Chemical Research Institute (Tianjin, China). Chlorpromazine, amiloride and methyl‐β‐cyclodextrin, 2‐mercaptoethanol, and bismuth chloride were obtained from Sigma Aldrich (Shanghai, China).DOX was bought from Beijing HuaFeng United Technology Co., Ltd. (Beijing, China). 100 kDa cutoff ultrafiltration membrane was purchased from Millipore (Bedford, USA). BSA (66 kDa), antibiotics penicillin (100 U mL^−1^) and streptomycin (100 µg mL^−1^) were obtained from Biosharp Co., Ltd (Hefei, China). RPMI 1640 culture medium, PBS, 0.25% TE and FBS were bought from Gibco (Grand Island, USA). Cell Counting Kit‐8 (CCK‐8) was bought from Dojindo Laboratories (Kumamoto, Japan). LysoTracker Deep Red was purchased from Thermo Fisher Scientific (Waltham, USA). DiO and calcein‐AM and PI were obtained from Shanghai Yisheng Biological Technology Co., Ltd. (Shanghai, China). DI water was prepared from Millipore (Bedford, USA). VAMP 2, VAMP 3, VAMP 7, and CD54 rabbit monoclonal Ab were bought from Proteintech Group, Inc. (Wuhan, China). All the other reagents were obtained from Sigma Aldrich (Shanghai, China).

*Synthesis of Bi_2_Se_3_ Nanodots*: The Bi_2_Se_3_ nanodots were synthesized according to previous work with a minor modification.^52^ Firstly, 0.3 mmol selenium powder was dissolved in a mixture of 2.85 mmol 2‐mercaptoethanol and 9.9 mmol ethylenediamine. The mixture solution was added into 150 mL BSA aqueous solution (2 mg mL^−1^) under drastic magnetic stirring in a 250 mL round‐bottomed flask. And then 10 mL hydrochloric acid (1.2 mol L^−1^) solution with 0.2 mmol bismuth chloride was quickly added to the above mentioned round‐bottomed flask. After stirring for 2 h at room temperature, the black solution was centrifuged for 10 min at 10 000 × *g*, the obtained colloidal supernatant was further washed and concentrated using a 100 kDa cutoff ultrafiltration membrane. The final Bi_2_Se_3_ nanodot solution was lyophilized and stored at 4 °C.

*Preparation of Bi_2_Se_3_/DOX@MPs*: 1 × 10^7^ murine H22 hepatocellular carcinoma cells were intraperitoneally (i.p.) injected into BALB/c mice. After a week, a plenty of H22 cells were obtained from hepatocellular carcinoma ascites. The obtained H22 cells were further used for the generation of MPs.

To load the H22 cells with Bi_2_Se_3_ nanodots and DOX, Bi_2_Se_3_ nanodots (400 µg mL^−1^) and DOX (100 µg mL^−1^) were mixed in serum‐free RPMI 1640 medium, suspending the H22 cells at a concentration of 2.50 × 10^6^ cells mL^−1^. Then 400 µL cell/Bi_2_Se_3_/DOX mixture were added into the 0.4 cm electroporation cuvettes, and incubated for 10 min at 4 °C before electroporation. The electroporation process was carried out at 300 V and 150 µF using a Bio‐Rad Gene Pulser Xcell electroporation system, the final mixture was incubated at 37 °C for 2 h to allow the recovery of the plasma membrane of the H22 cells. Different voltages (100, 200, 300, 400 V), DOX concentrations (25, 50, 100, 200 µg mL^−1^) or Bi_2_Se_3_ concentrations (100, 200, 400, 800 µg mL^−1^) were proceeded as described above to evaluate the controllability of the electroporation.

In order to trigger vesicle release from donor cells, Bi_2_Se_3_/DOX loaded H22 cells were exposed to ultraviolet irradiation (UBV, 300 J m^−2^) for 1 h. After 12 h, supernatants were centrifuged for 10 min at 2000 × *g* to get rid of dead cells and cell debris. At last, the resulting supernatants were further centrifuged for 60 min at 20 000 × *g* to isolate and concentrate Bi_2_Se_3_/DOX@MPs. The obtained Bi_2_Se_3_/DOX@MPs were washed with sterile PBS and resuspended in culture medium for the following experiments. The stability of Bi_2_Se_3_/DOX@MPs stored in PBS and FBS at 4 °C for 7 days was real‐day monitoring by a Zetasizer Nano ZS90 (Malvern, UK).

*Characterization of Bi_2_Se_3_/DOX@MPs*: For TEM characterization, MPs and Bi_2_Se_3_/DOX@MPs were fixed in a 5% glutaraldehyde solution, and staining with 2% phosphotungstic acid. Then samples were dropped onto a copper grids coated carbon membrane, and observed using a HT7700 microscope (Hitachi, Japan) at 80 kV. STEM‐EDS line‐scan analysis of Bi_2_Se_3_/DOX@MPs was performed using a Talos F200X atomic resolution analytical microscope (FEI, The Netherlands). Dynamic light scattering (DLS) and zeta potential of samples were measured on a Zetasizer Nano ZS90 (Malvern, UK). The protein content of the MPs was determined with using the BCA protein assay kit (Beyotime, China) according to the manufacturer's protocol. The UV–vis–NIR absorption spectra of Bi_2_Se_3_/DOX@MPs at different concentrations were acquired on a TU‐1901 UV–vis spectrophotometer (Beijing Purkinje General Instrument Co., Ltd.) at room temperature. The fluorescence spectra of MPs, DOX, and Bi_2_Se_3_/DOX@MPs were measured by using a FluoroMax‐4 spectrofluorometer (HORIBA, USA). Dark‐field optical microscopy images were recorded using an inverted fluorescence microscope (Nikon Eclipse TiS, USA) observed with a highly numerical dark‐field condenser.

For fluorescence colocalization analysis, MPs and Bi_2_Se_3_/DOX@MPs were incubated in the presence of fluorescent dyes DiO (10 × 10^−6^ m) for 30 min at 37 °C. Then the mixture were centrifuged at 20 000 × *g* for 60 min to remove free dye and washed by PBS for three times. The fluorescence images of DiO‐labeled MPs, Bi_2_Se_3_/DOX@MPs, and DiO‐labeled Bi_2_Se_3_/DOX@MPs were performed with a FV1000‐IX81 CLSM (OLYMPUS, Japan).

To compare the encapsulation efficiency of Bi_2_Se_3_ nanodots and DOX, the H22 cells were incubated with the Bi_2_Se_3_ nanodots and DOX at 37 °C for 2 h, then Bi_2_Se_3_/DOX@MPs was obtained under the same operation as described above. The donor cells and Bi_2_Se_3_/DOX@MPs obtained by incubation or electroporation were digested with hydrogen nitrate and perchloric acid. Then the content of bismuth was determined by AFS‐930 atomic fluorescence spectrometer (Titan, China). The DOX concentration was measured using spectrofluorometer (Ex. 488 nm, Em. 580 nm). The DOX and Bi_2_Se_3_ loading contents of Bi_2_Se_3_/DOX@MPs generated from donor cells by electroporation or incubation were detected by the same method. For in vitro drug release study, the cumulative amounts of DOX release studies were performed in pH 7.4 PBS buffer at 37 °C by the dialysis method.

For SDS‐PAGE protein analysis, reduced proteins (10–25 µg) of H22 cells, unloaded MPs and Bi_2_Se_3_/DOX@MPs were loaded into a 12% Bis‐Tris gel and run at 110 V by a DYY‐7C electrophoresis system (Liuyi Instrument, Beijing, China). SDS‐PAGE ruler prestained protein ladder (Thermo Fisher, Waltham, USA) was used to track protein migration. The resulting gels were stained with Coomassie blue to identify the proteins.

*Photothermal Property Study*: The photothermal properties of different concentrations (Bi_2_Se_3_ concentration, 0.00, 12.5, 25.0, 50.0, and 100 µg mL^−1^) of Bi_2_Se_3_/DOX@MPs were measured upon irradiation of an 808 nm NIR laser (BWT Beijing Ltd.) (1.5 W cm^−2^, 10 min), and the temperature and infrared thermal images at different time points were recorded by an infrared thermal imaging camera (FLIR E8, USA). The photothermal performances of Bi_2_Se_3_ nanodots, Bi_2_Se_3_ @MPs, and Bi_2_Se_3_/DOX@MPs at Bi_2_Se_3_ concentration of 12.5, 25.0, 50.0, and 100 µg mL^−1^ were studied as above‐mentioned condition. To estimate the photothermal stability of Bi_2_Se_3_/DOX@MPs, the samples were irradiated with an 808 nm NIR laser (1.5 W cm^−2^, 10 min), then naturally cooling to room temperature without irradiation, four cycles of alternating heating and cooling was repeated.

*Cell Culture*: Murine hepatocellular carcinoma cell line H22, human hepatocellular carcinoma cell line Bel7402, human umbilical vein endothelial cells (HUVEC), and mouse mammary tumor cells 4T1 were obtained from China Center for Type Culture Collection (CCTCC). Cells were cultured in RPMI 1640 culture medium supplemented with 10% FB, antibiotics penicillin (100 U mL^−1^) and streptomycin (100 µg mL^−1^) at 37 °C in the presence of 5% CO_2_.

*Cellular Uptake Assay*: H22 cells were seeded in 6‐well plates at a density of 1.5 × 10^5^ cells per well overnight. The culture medium was replaced with fresh serum‐free RPMI 1640 culture medium containing free DOX, Bi_2_Se_3_ nanodots or Bi_2_Se_3_/DOX@MPs at the concentration of 1.00 µg mL^−1^ DOX or 5.5 µg mL^−1^ Bi_2_Se_3_ and cells were incubated for 2 and 4 h. Then, the cells were rinsed with PBS three times, and the quantification of intracellular fluorescence intensity of DOX was measured by FC500 flow cytometry (Beckman, USA) with argon laser excitation at 488 nm and fluorescence (FL2) detection. Then the content of bismuth was determined by AFS‐930 atomic fluorescence spectrometer (Titan, China). In addition, to obtain the intracellular fluorescence images, H22 cells were incubated with free DOX or Bi_2_Se_3_/DOX@MPs for 2 and 4 h as aforementioned method, then treated with 4% paraformaldehyde solution and stained with 4,6‐ diamidino‐2‐penylindole (DAPI) (5 µg mL^−1^) for 10 min. The cells were observed by the FV1000‐IX81 CLSM. In order to verify the outstanding uptake capacity of H22 cells for Bi_2_Se_3_/DOX@MPs, BEL‐7402, HUVEC, and 4T1 cells were also chosen to incubate with Bi_2_Se_3_/DOX@MPs at the same concentration for 2, 4, and 8 h in vitro. The cellular uptakes of Bi_2_Se_3_/DOX@MPs were detected by flow cytometry.

To investigate the cellular internalization pathways of Bi_2_Se_3_/DOX@MPs, H22 or Bel7402 cells were incubated with Bi_2_Se_3_/DOX@MPs for 2 and 4 h. After rinsing in PBS, the H22 or Bel7402 cells were incubated with LysoTracker Deep Red (1000 times dilution in PBS) for lysosome staining. Then the H22 or Bel7402 cells were treated with 4% paraformaldehyde solution and stained with DAPI (5 µg mL^−1^) for 10 min. Finally, the cells were observed by the FV1000‐IX81 CLSM.

Furthermore, Bi_2_Se_3_/DOX@MPs was incubated in the presence of fluorescent dyes DiO (10 × 10^−6^ m) for 30 min at 37 °C, then DiO‐labeled Bi_2_Se_3_/DOX@MPs was obtained after centrifugation. As stated above, H22 or Bel7402 cells were incubated with DiO‐labeled Bi_2_Se_3_/DOX@MPs for 2 and 4 h. After 10 min incubation, the H22 or Bel7402 cells intracellular fluorescence images were obtained as aforementioned method.

To further confirm the cellular internalization pathways of Bi_2_Se_3_/DOX@MPs, H22 cells were preincubated with endocytosis inhibitors chlorpromazine (10 µg mL^−1^), amiloride (50 µg mL^−1^), and methyl‐β‐cyclodextrin (2 mg mL^−1^) for 1 h and then incubated with Bi_2_Se_3_/DOX@MPs for 6 h. Besides, H22 cells also incubated with Bi_2_Se_3_/DOX@MPs at 4 °C for 6 h. The cellular uptakes of Bi_2_Se_3_/DOX@MPs were detected by flow cytometry.

To study the relationship between cellular uptake and SNARE proteins, Bi_2_Se_3_/DOX@MPs was pretreated with VAMP 2, VAMP 3, VAMP 7 or VAMP 2/3/7 rabbit monoclonal Ab (1:200) for 1 h at room temperature before incubating with H22 cells. Besides, to study the effects of calcium ions, 200 × 10^−6^ m Ca^2+^ was added when incubating with H22 cells. The cellular uptakes of treated or untreated Bi_2_Se_3_/DOX@MPs were detected by flow cytometry.

The fluorescence of LysoTracker Deep Red, DOX, DiO, and DAPI were observed using a 650/670, 559/580 nm, 488/520 nm, and 405/450 nm excitation/emission filter, respectively.

*Penetration of Bi_2_Se_3_/DOX@MPs into H22 3D Tumor Spheroids*: H22 3D tumor spheroids were constructed as previous report.^55^ The fibrinogen/cell mixtures were obtained by blending H22 cell solution (3.2 × 10^4^ cells mL^−1^) with the same volume of fibrinogen (2 mg mL^−1^). Then 50 µL mixtures were added into each well of the 96 well plates preadded with 1 µL of thrombin (0.1 U µL^−1^). At last, 200 µL of RPMI 1640 culture medium was added into each well of the 96 well plates, and incubated at 37 °C with 5% CO_2_. After five days, the diameters of H22 3D tumor spheroids were estimated to grow to about 100–150 µm.

To investigate the penetration of Bi_2_Se_3_/DOX@MPs into H22 3D tumor spheroids, free DOX or Bi_2_Se_3_/DOX@MPs at the concentration of 2 mg mL^−1^ DOX were treated with H22 3D tumor spheroids for 4 h, respectively. The H22 3D tumor spheroids were rinsed with PBS for three times, fixed with 4% paraformaldehyde for 30 min. Finally, the DOX fluorescence images were observed using Z‐stack imaging with 5 µm spaces from the top of the tumor spheroids by the CLSM with a 488/580 nm excitation/emission filter.

*Cell Viability Assay*: H22 cells were seeded in 96 well plates at a density of 1 × 10^4^ cells per well and incubated for 12 h. Then the cells were treated with free DOX, Bi_2_Se_3_ nanodots, Bi_2_Se_3_@MPs, and Bi_2_Se_3_/DOX@MPs at DOX concentrations from 0.625 to 5.00 µg mL^−1^ or Bi_2_Se_3_ concentrations from 3.438 to 27.5 µg mL^−1^ for 24 h without irradiation. Another treated 96 well plates with the same protocols intended to evaluate the photothermal toxicity were incubated for 4 h firstly, then irradiated by 808 nm laser with a power density of 1.5 W cm^−2^ for 5 min and incubated for another 20 h. After the treatment above, 10 µL of CCK‐8 solution was added into per well and its absorbance was measured at 450 nm to calculate the percentage of viability of cells.

Calcein‐AM and PI double staining experiment was also carried out to evaluate the cell activity. Firstly, 1 × 10^5^ cells were incubated with Bi_2_Se_3_/DOX@MPs for 4 h, and then the cells irradiated with an 808 nm laser with a power density of 1.5 W cm^−2^ for 5 min and incubated for another 20 h. Finally, the cells were incubated with calcein‐AM and PI for 15 min at 37 °C. Live and dead cells were stained by Calcein AM (green fluorescence) and PI (red fluorescence).

*Tumor Mouse Model*: Male BALB/c mice were purchased from the Center for Disease Control and Prevention in Hubei Province, China. All mice were housed in a room with controlled temperature for at least one week prior to study. All animal studies were performed according to the guidelines approved by the Institutional Animal Care and Use Committee at Tongji Medical College, Huazhong University of Science and Technology (HUST, Wuhan, China). The H22 tumor‐bearing BALB/c mice were generated by subcutaneous injection of 2 × 10^6^ H22 cells in 100 µL PBS into the backside of male BALB/c mice. Tumors were allowed to grow for 6–8 days, reaching about 80–100 mm^3^, before further studies were performed.

*Hemolysis Assay of Bi_2_Se_3_/DOX@MPs In Vitro*: The hemolysis assay of Bi_2_Se_3_/DOX@MPs in vitro was measured to evaluate the blood compatibility. Heparin sodium‐stabilized mice blood samples were freshly collected from BALB/c mice. Firstly, 1 mL of blood sample was added to 2 mL of physiological saline, and then the serum was removed from erythrocyte cells by centrifugation at 3000 rpm for 10 min, the erythrocyte cells were further washed several times to ensure the removal of any released hemoglobin and resuspended in 5 mL physiological saline. DI water and physiological saline incubation with erythrocyte cells at room temperature for 2 h was used as the positive (+) and negative (−) control, respectively. The Bi_2_Se_3_/DOX@MPs at different Bi_2_Se_3_ concentrations of 10, 25, 50, 100, and 200 µg mL^−1^ were incubated with erythrocyte cells suspension at room temperature for 2 h. At last, the mixtures were centrifuged at 3000 rpm for 10 min, and the absorbance values of the supernatants at 575 nm were determined by using UV–vis absorption spectrum. The hemolytic activity percentage of the Bi_2_Se_3_/DOX@MPs was calculated as follows

(1) $$\mathrm{Hemolysis}\;\%=\frac{A_{\mathrm{sample}}-A_{\mathrm{negative}\;\mathrm{control}}}{A_{\mathrm{positive}\;\mathrm{control}}-A_{\mathrm{negative}\;\mathrm{control}}}\times 100\%$$

*In Vivo Biodistribution and Photothermal Performance*: For biodistribution studies, H22 tumor‐bearing BALB/c mice (*n* = 3 each group) were intravenous injected with Bi_2_Se_3_ nanodots and Bi_2_Se_3_/DOX@MPs at Bi_2_Se_3_ dose of 26 mg kg^−1^ via tail vein and sacrificed at different time points (6, 12, 24, and 48 h). To study the homing target capability, Bi_2_Se_3_/DOX@MPs were pretreated with CD54 rabbit monoclonal Ab (1:200) for 1 h at room temperature or 0.25% TE for 10 min at 37 °C before injection, the 12 h biodistribution were studied. Major organs (heart, liver, spleen, lung, kidney) and tumors were collected and rinsed with physiological saline. The content of bismuth in major organs and tumors was obtained using the above‐mentioned method. The concentration of bismuth in tissues was expressed as percentage of injected dose per gram of tissue (% ID g^−1^).

To evaluate the photothermal performance of Bi_2_Se_3_/DOX@MPs in vivo, the H22 tumor‐bearing BALB/c mice were treated with PBS, Bi_2_Se_3_ nanodots, and Bi_2_Se_3_/DOX@MPs at a Bi_2_Se_3_ dose of 6.6 mg kg^−1^ via intravenous injection. At 12 h post‐injection, the tumors were irradiated with the 808 nm NIR laser (1.5 W cm^−2^, 10 min), the temperature of tumors and photothermal images were recorded at different time points (0, 2, 4, 6, 8, 10 min).

*In Vitro and In Vivo CT/PA Imaging*: Firstly, Bi_2_Se_3_/DOX@MPs solutions with different Bi_2_Se_3_ concentrations of 0, 0.0875, 0.35, 0.7, and 1.4 mg mL^−1^ were prepared for in vitro PA signals collection. Then, to evaluate the PA imaging performance in vivo, the H22 tumor‐bearing BALB/c mice with tumor size of 120–150 mm^3^ were injected with Bi_2_Se_3_/DOX@MPs (20 mg kg^−1^) via the tail vein. After the injection, the PA images of tumor regions in the injected mice were acquired at different time points (0, 1, 6, 12, and 24 h) and the average PA‐signal intensity value of the tumor regions was measured. 808 nm wavelength of excitation light was used to collect the PA signals.

In addition, Bi_2_Se_3_/DOX@MPs and iohexol were prepared with the same Bi/I ions concentrations (0, 1.75, 3.5, 7, 14, and 28 × 10^−3^ m) for CT scans in vitro firstly. The H22 tumor‐bearing BALB/c mice with tumor size of 120–150 mm^3^ were intratumorally injected with Bi_2_Se_3_/DOX@MPs (28 × 10^−3^ m, 50 µL) for CT imaging in vivo.

*In Vivo Antitumor Activity of Combination Therapies*: H22 tumor‐bearing BALB/c mice were prepared as described above. When the tumor volume reached about 80–100 mm^3^, the H22 tumor‐bearing BALB/c mice were randomly assigned to seven groups, and respectively intravenously injected with 200 µL PBS, PBS+NIR, free DOX, Bi_2_Se_3_ nanodots+NIR, Bi_2_Se_3_@MPs+NIR, Bi_2_Se_3_/DOX@MPs and Bi_2_Se_3_/DOX@MPs+NIR in a single dose of 1.20 mg kg^−1^ of DOX or 6.6 mg mg kg^−1^ of Bi_2_Se_3_. After 12 h post‐injection, the tumors were irradiated with or without 808 nm NIR irradiation (1.5 W cm^−2^, 10 min). Body weight and tumor size of each group were measured every day. The tumor volume (*V*, mm^3^) was calculated to be *V* = *ab* ^2^/2, where *a*, *b* represent the length and width of tumors, respectively.

*Histological Analysis*: The histological analysis was conducted after 15 days treatment. Mice were sacrificed and their tumors and major organs including heart, liver, spleen, lung, and kidney were harvested. Then, the excised tissues were fixed with 4% paraformaldehyde, sectioned, and stained with H&E for histological analysis.

*Blood Chemistry Assays*: After 7 days treatment, the blood samples of each mouse were collected and centrifuged at 3000 rpm for 10 min at 4 °C to obtain plasma samples for measuring clinical parameters. Aminotransferase (ALT), aspartate aminotransferase (AST), creatine kinase (CK), dehydrogenase (LDH) and blood urea nitrogen (BUN) were measured on a Beckman Coulter AU680 analyzer (Beckman Coulter, Miami, FL, USA).

*Statistical Analysis*: All experiments were repeated at least three times. Data were analyzed by Student's *t*‐test, *p‐values of <0.05 were considered significant, and **p‐values of <0.01 were considered highly significant.

## Conflict of Interest

The authors declare no conflict of interest.

## Supporting information

Supporting Information
