# Supplementary material for: Engineered Cell‐Derived Microparticles Bi2Se3/DOX@MPs for Imaging Guided Synergistic Photothermal/Low‐Dose Chemotherapy of Cancer
Source: Adv Sci (Weinh). 2019 Dec 12;7(3):1901293. doi: 10.1002/advs.201901293 (PMC7001653; doi:10.1002/advs.201901293)
Supplement: Supplementary file 1 — Supporting Information [file ADVS-7-1901293-s001.pdf]

Copyright WILEY-VCH Verlag GmbH & Co. KGaA, 69469 Weinheim, Germany, 2019.

ADVANCED SCIENCE
Open Access

Supporting Information

for *Adv. Sci.,* DOI: 10.1002/advs.201901293

Engineered Cell-Derived Microparticles Bi_2Se_3/DOX@MPs for Imaging Guided Synergistic Photothermal/Low-Dose Chemotherapy of Cancer

Dongdong Wang, Yuzhu Yao, Junkai He, Xiaoyan Zhong, Basen Li, Shiyu Rao, Haiting Yu, Shuaicheng He, Xiaoyu Feng, Tuo Xu, Bin Yang, Tuying Yong, Lu Gan, Jun Hu, and Xiangliang Yang**

Copyright WILEY-VCH Verlag GmbH & Co. KGaA, 69469 Weinheim, Germany, 2019.

Supporting Information

Engineered Cell-Derived Microparticles Bi_2Se_3/DOX@MPs for Imaging Guided Synergistic Photothermal/Low-Dose Chemotherapy of Cancer

Dongdong Wang, Yuzhu Yao, Junkai He, Xiaoyan Zhong, Basen Li, Shiyu Rao, Haiting Yu, Shuaicheng He, Xiaoyu Feng, Tuo Xu, Bin Yang, Tuying Yong, Lu Gan, Jun Hu, and Xiangliang Yang**

D. Wang, Y. Yao, J. He, X. Zhong, S. Rao, H. Yu, S. He, X. Feng, T. Xu, B. Yang, Dr. T. Yong, Prof. L. Gan, A/Prof. J. Hu and Prof. X. Yang
National Engineering Research Center for Nanomedicine
College of Life Science and Technology
Huazhong University of Science and Technology
Wuhan 430074, P. R. China
E-mail: hjun0718@hust.edu.cn; yangxl@hust.edu.cn
Dr. B. Li
Department of Radiology, Tongji Hospital
Tongji Medical College
Huazhong University of Science and Technology
Wuhan 430074, P.R. China

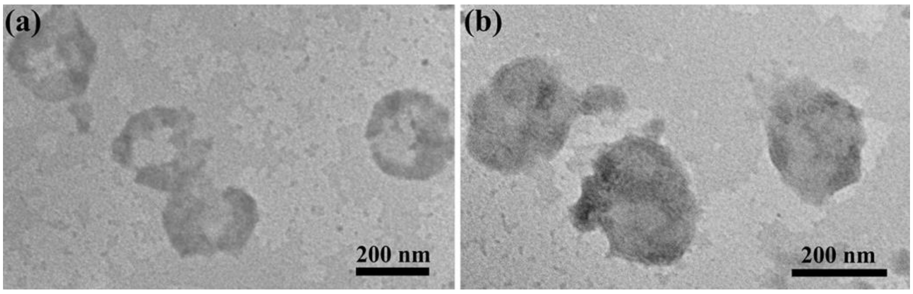

Figure S1. TEM images of multiple MPs (a) and Bi_2Se_3/DOX@MPs(b).

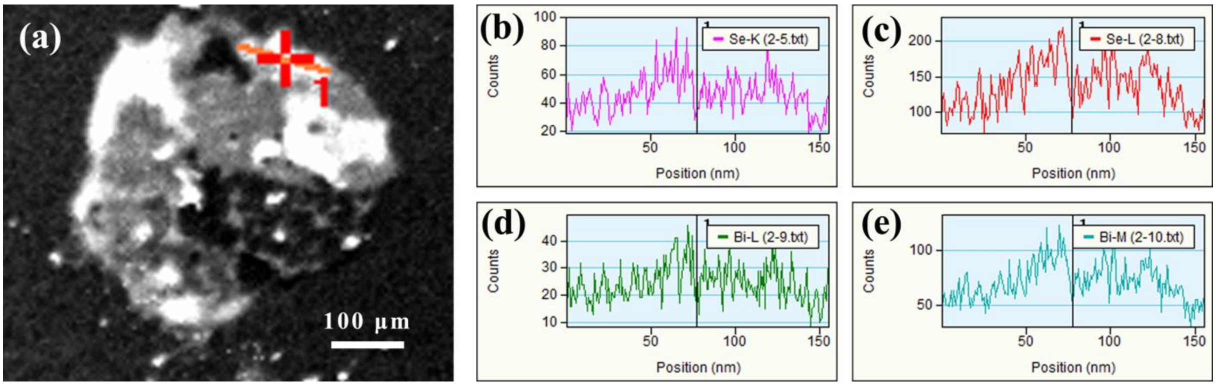

Figure S2. FTEM-EDS line-scan analysis of Bi_2Se_3/DOX@MPs.

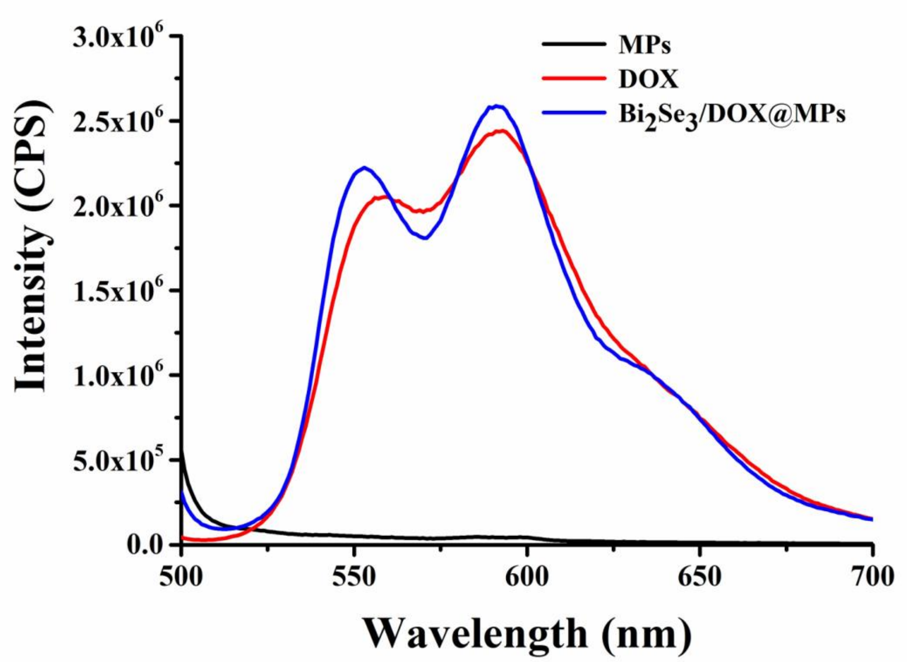

Figure S3. Fluorescence spectra of MPs, free DOX and Bi_2Se_3/DOX@MPs at the excitation of 488 nm.

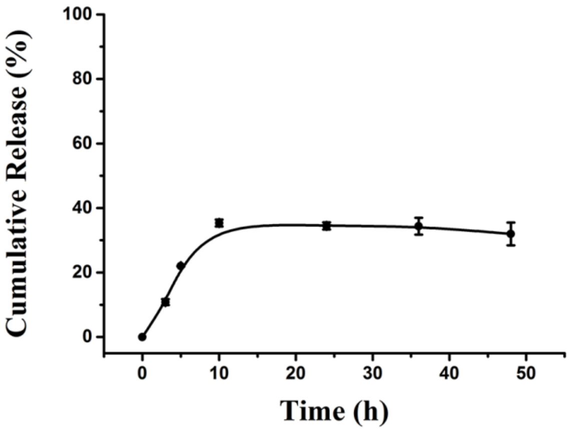

Figure S4. Cumulative amounts of DOX released from Bi_2Se_3/DOX@MPs in pH 7.4 PBS buffer at 37 °C.

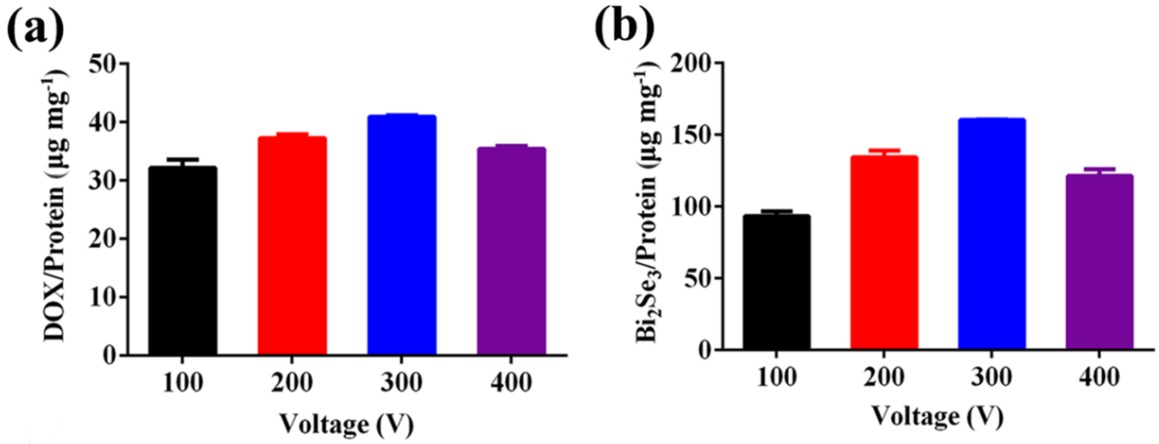

Figure S5. Voltage-dependent DOX (a) and Bi_2Se_3 (b) loading efficiency in Bi_2Se_3/DOX@MPs.

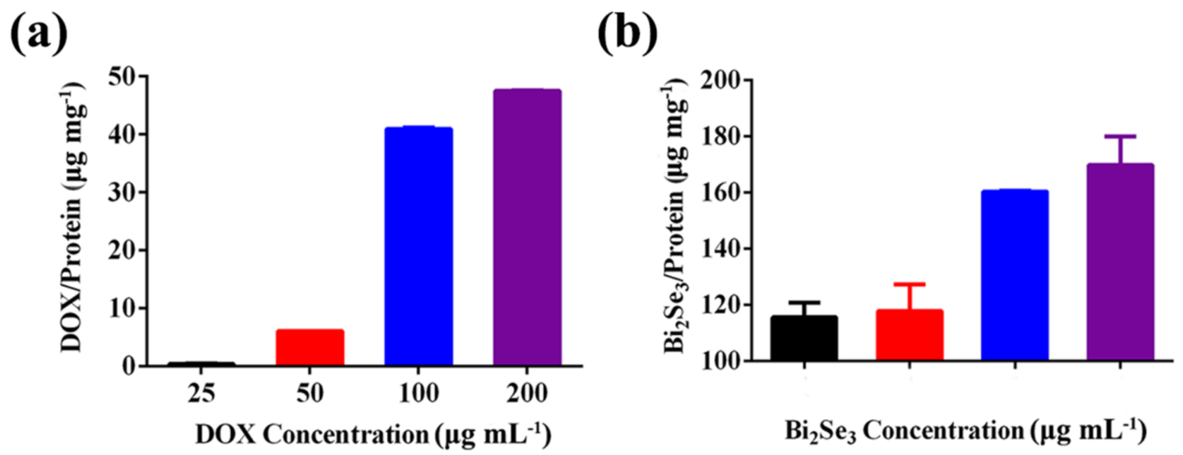

Figure S6. DOX and Bi_2Se_3 concentration-dependent loading efficiency in Bi_2Se_3/DOX@MPs.

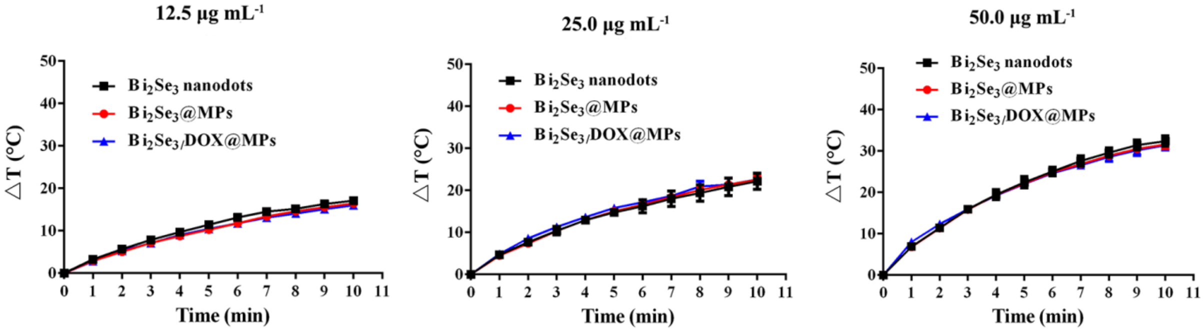

Figure S7. Comparison on temperature elevation of Bi_2Se_3 nanodots, Bi_2Se_3@MPs and Bi_2Se_3/DOX@MPs at different concentrations (12.5, 25, 50 μg mL^{-1}) under 808 nm NIR irradiation for 10 min.

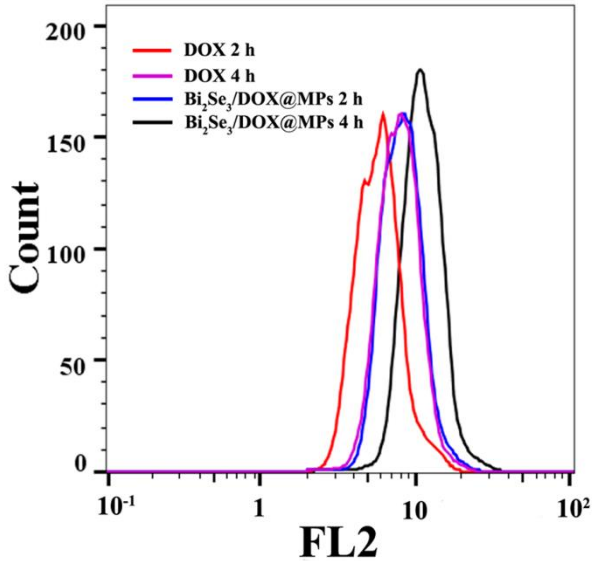

Figure S8. Flow cytometric profile of H22 cells respectively incubated with free DOX and Bi2Se3/DOX@MPs for 2 h and 4 h.

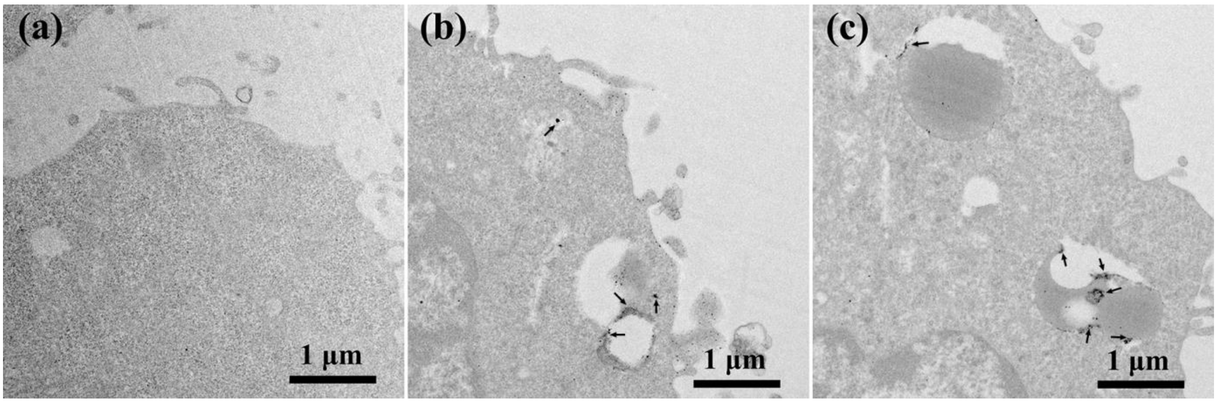

Figure S9. TEM images of control H22 cells (a) and H22 cells incubated with Bi_2Se_3/DOX@MPs for 2 h (b) or 4 h(c). The black arrows indicate Bi_2Se_3 nanodots.

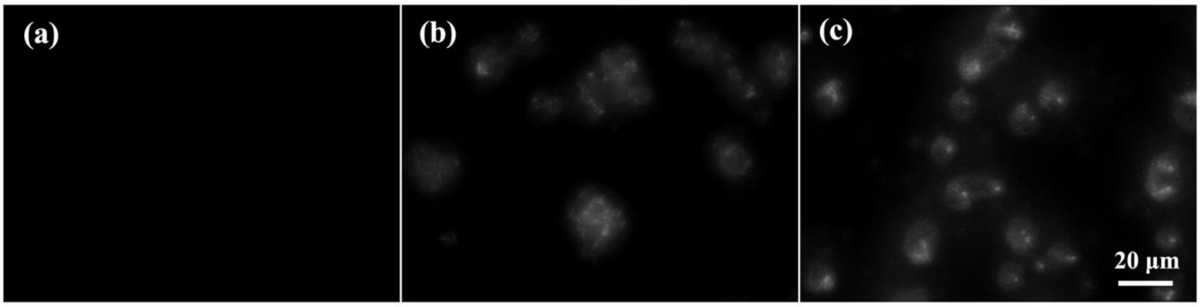

Figure S10. Dark-field optical microscopy images of control H22 cells (a) and H22 cells incubated with Bi_2Se_3/DOX@MPs for 2 h (b) or 4 h(c).

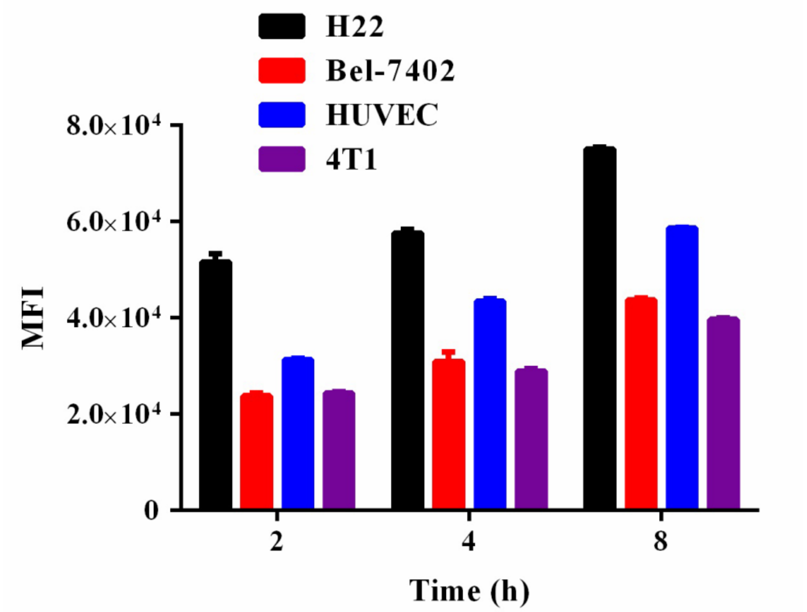

Figure S11. Cellular uptake of Bi_2Se_3/DOX@MPs incubated with different cells (H22, Bel-7402, HUVEC and 4T1 cells) for 2, 4 and 8 hours.

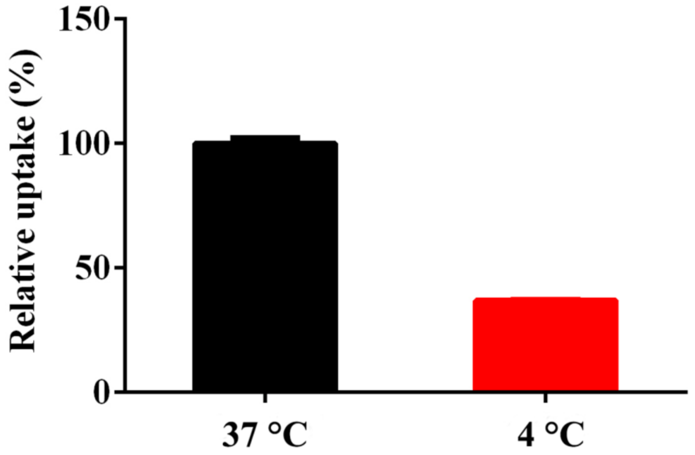

Figure S12. Relative cellular uptake of Bi_2Se_3/DOX@MPs incubated H22 cells at 37 °C and 4 °C.

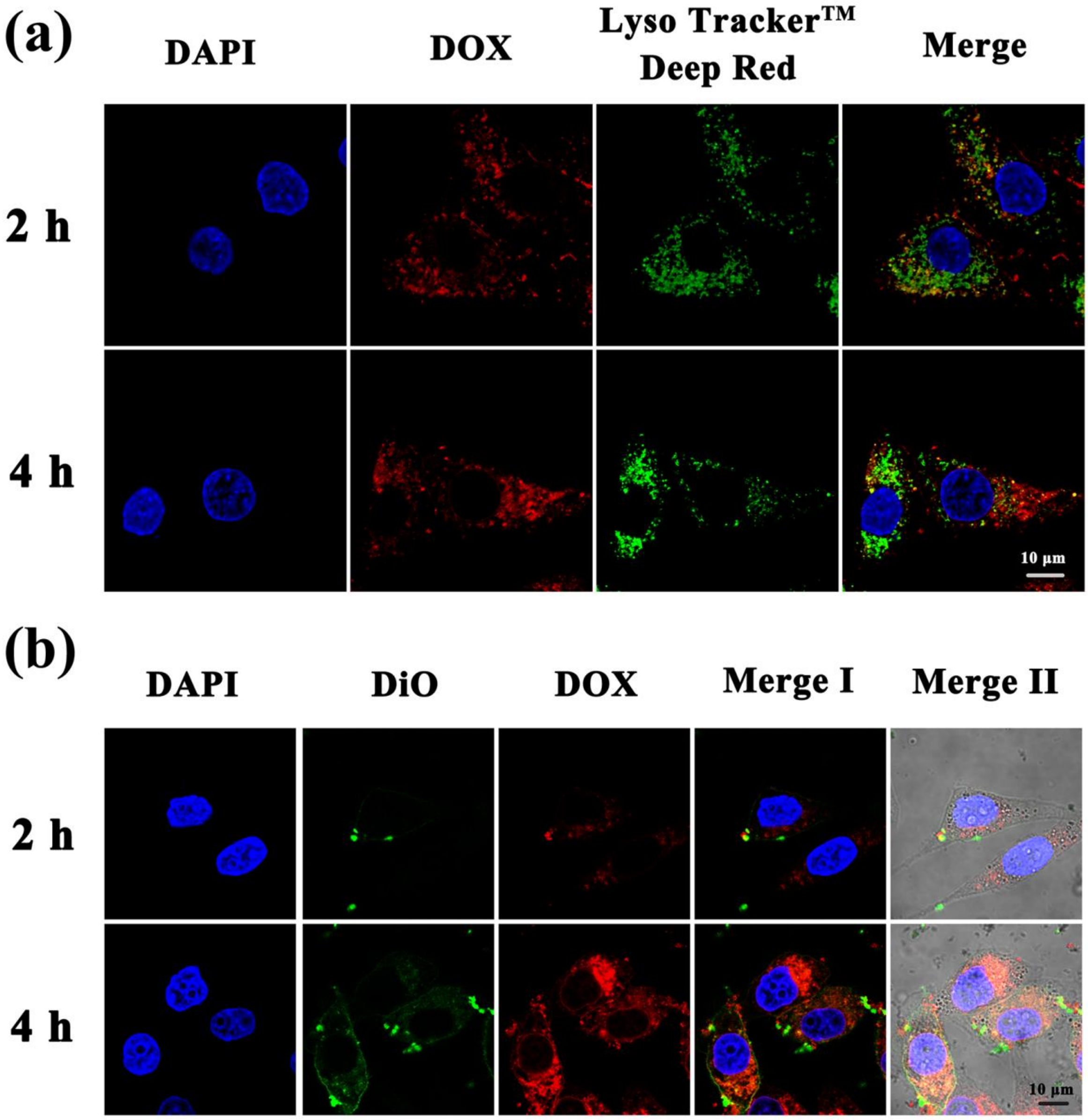

Figure S13. Cellular internalization pathway of Bi_2Se_3/DOX@MPs. CLSM images of Bel 7402 cells incubated with Bi_2Se_3/DOX@MPs (a) and DiO-labled Bi_2Se_3/DOX@MPs (b) for 2 and 4 h. Cell nucleus were labeled with DAPI (blue) in both a and b. Lysosomes were labeled with LysoTracker Deep Red (green) in a. The concentration of DOX was fixed at 1 μg mL^{-1}.

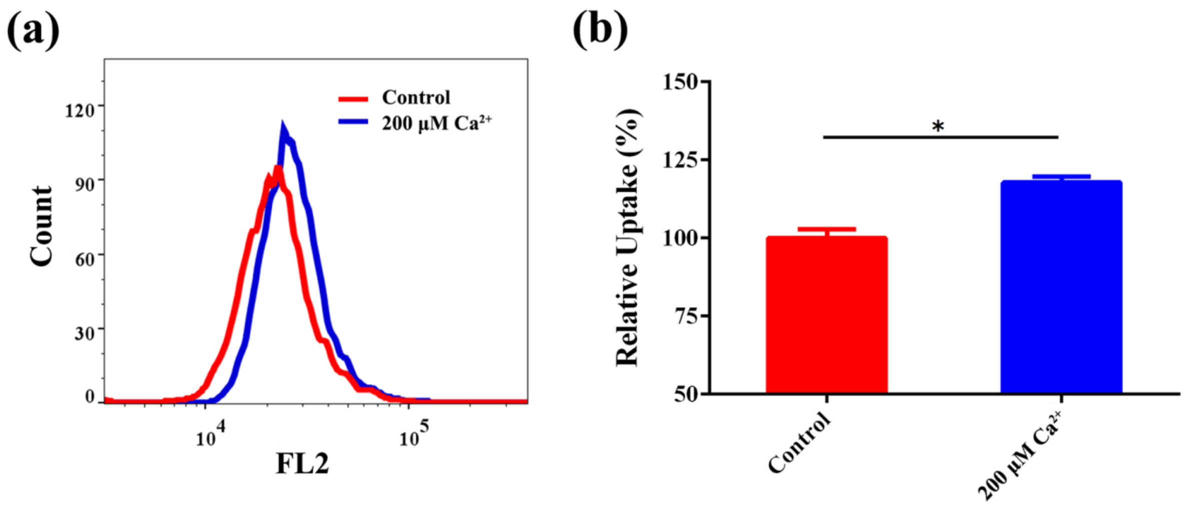

Figure S14. Flow cytometric profile (a) and relative cellular uptake (b) of Bi_2Se_3/DOX@MPs with or without 200 μM Ca^{2+}.

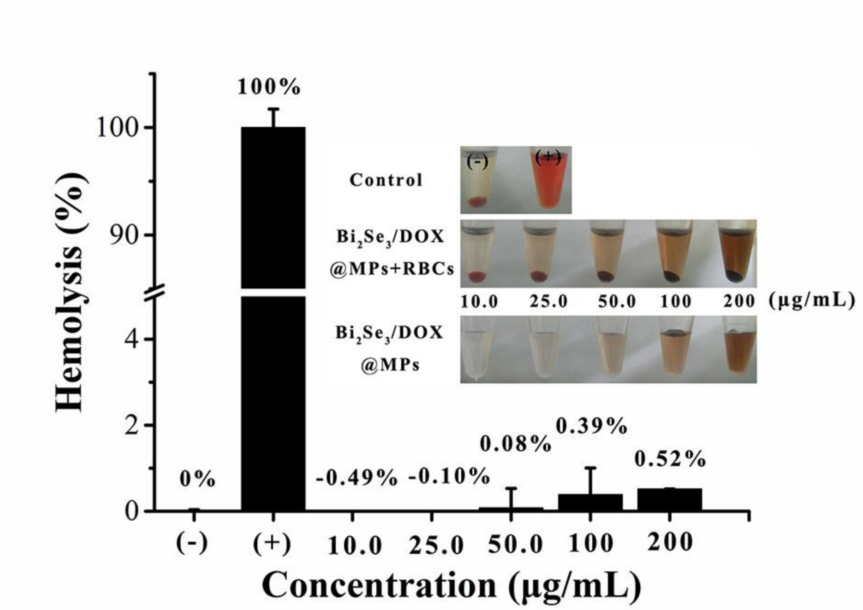

Figure S15. Hemolysis percentages of Bi_2Se_3/DOX@MPs in physiological saline at concentrations of 10, 25, 50, 100 and 200 μg mL^{-1}, inset is the photograph of control (up), hemolysis assay to detect the presence of hemoglobin in the supernatant of Bi_2Se_3/DOX@MPs (middle) and the Bi_2Se_3/DOX@MPs physiological saline solutions (down).

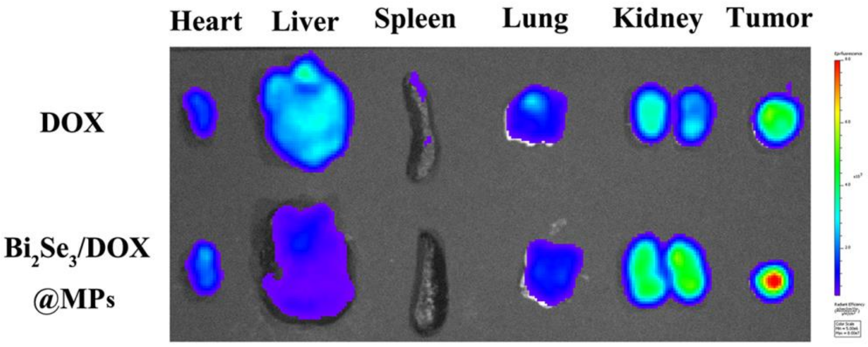

Figure S16. Ex vivo fluorescence images of heart, liver, spleen, lung, kidney, and tumor 12 h after treatment with free DOX and Bi_2Se_3/DOX@MPs.

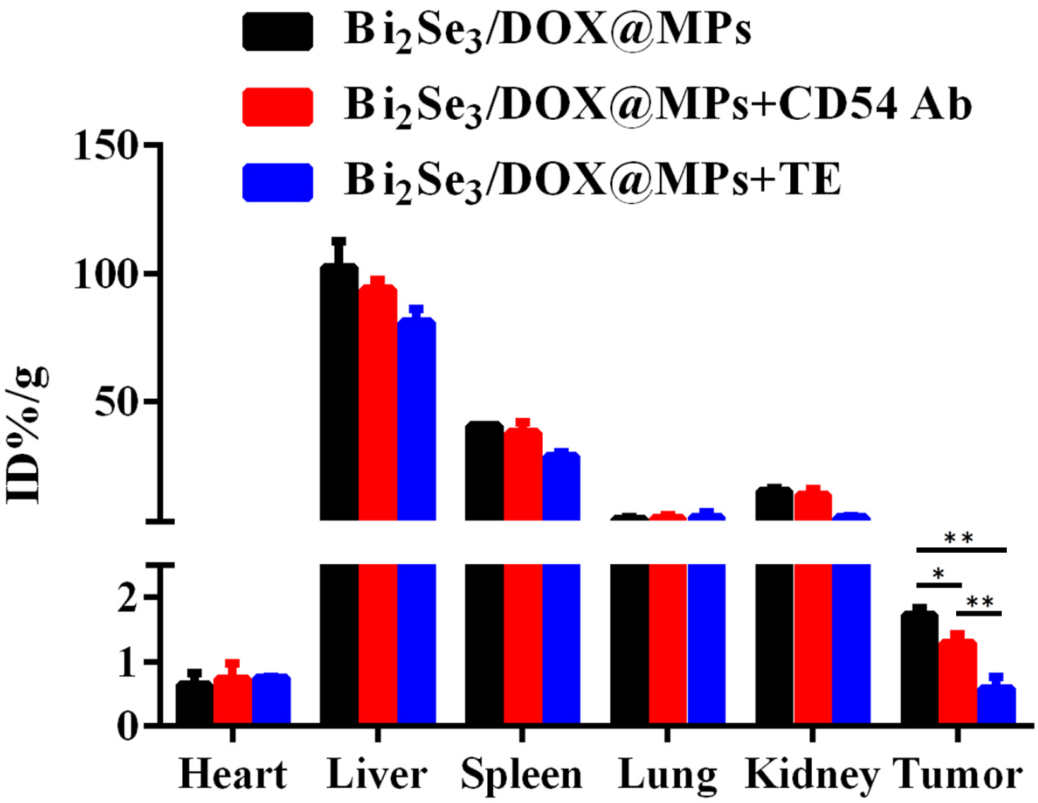

Figure S17. *In vivo* biodistribution of Bi_2Se_3/DOX@MPs before or after treating with CD54 Ab or 0.25 % TE.

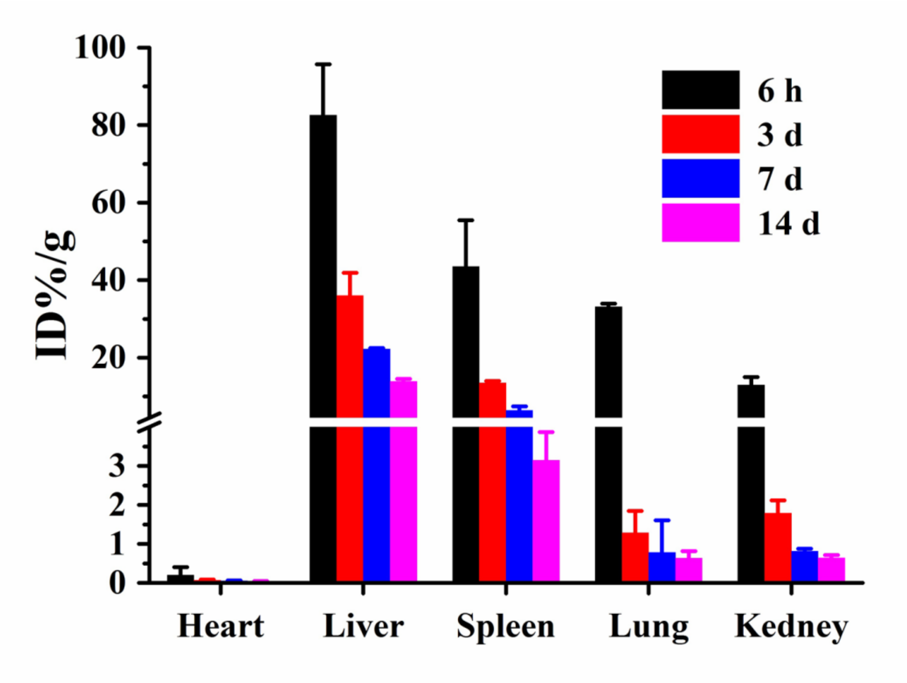

Figure S18. *In vivo* clearance of Bi element after injecting Bi_2Se_3/DOX@MPs intravenously at 6h, 3 d, 7 d and 14 d.

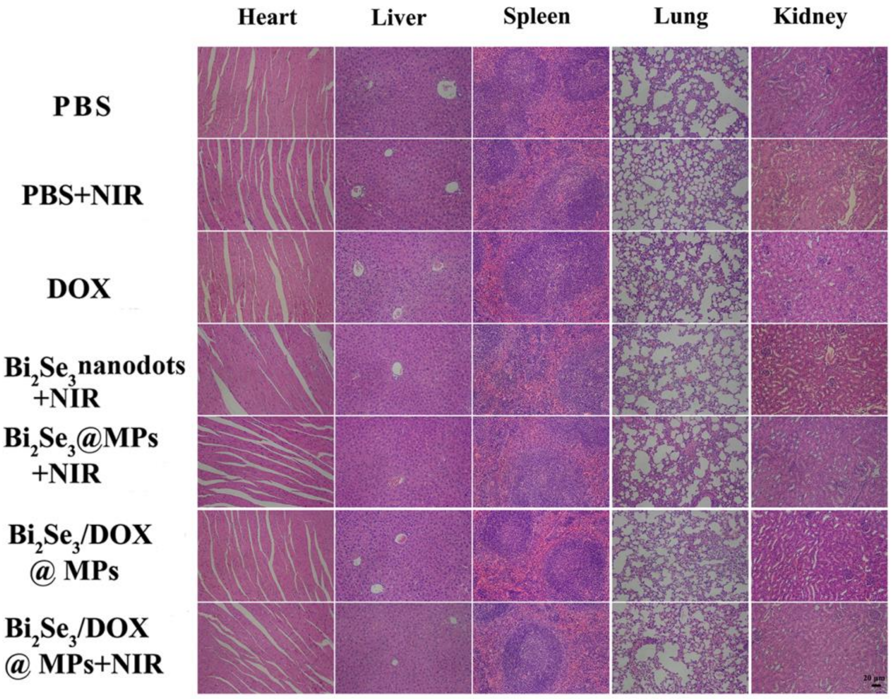

Figure S19. H&E staining images of heart, liver, spleen, lung, and kidney after the mice were sacrificed at 15st day post intravenous injection with PBS, free DOX, Bi_2Se_3 nanodots, Bi_2Se_3/@MPs and Bi_2Se_3/DOX@MPs with or without NIR irradiation. DOX and Bi_2Se_3 dosage were fixed at 1.2 and 6.6 mg kg^{-1}. Magnification 200×.

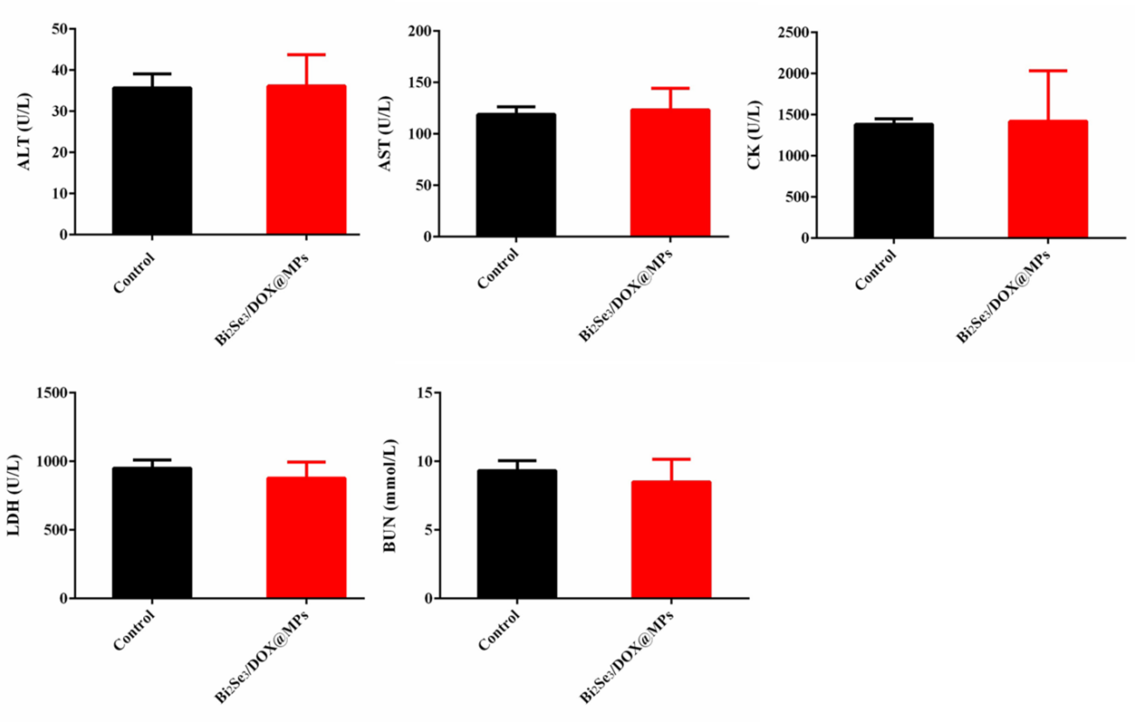

Figure S20. ALT, AST, CK, LDH and BUNs levels in the blood at day 7 after Bi_2Se_3/DOX@MPs treatment.
